# Stabilized Multicolor CsPbBr_3–*x*_I_*x*_ Nanocrystals via Ca–I Scorpionate Capping for Down-Light Converters

**DOI:** 10.1021/acsaom.6c00102

**Published:** 2026-04-14

**Authors:** Bárbara Nicoleth Vallejos, Ignacio Utreras-Asenjo, Enrique Francés-Poveda, Felipe de la Cruz-Martínez, Juwon Jang, Harumi Correa-Leiva, Víctor Mayorga, Oscar A. Douglas-Gallardo, Francisca Werlinger, Javier Martínez, Camilo Segura, Jhonatan Rodriguez-Pereira, Beatriz Julián-López, Seog Joon Yoon, Iván Mora-Seró, Carina Pareja-Rivera, Agustín Lara-Sánchez, Andrés F. Gualdrón-Reyes

**Affiliations:** † Facultad de Ciencias, Instituto de Ciencias Químicas, 28040 Universidad Austral de Chile, Isla Teja, Valdivia 5090000, Chile; ‡ Departamento de Química Inorgánica Orgánica y Bioquímica-Centro de Innovación en Química Avanzada (ORFEO−CINQA), Facultad de Ciencias y Tecnologías Químicas and Instituto Regional de Investigación Científica Aplicada-IRICA, 16733 Universidad de Castilla-La Mancha, 13071 Ciudad Real, Spain; § Department of Chemistry, College of Natural Science, 35032 Yeungnam University, Gyeongsan 38541, Republic of Korea; ∥ Departamento de Química Orgánica, Facultad de Química, 28056 Universidad de Concepción, Edmundo Larenas 129, Concepción 4070371, Chile; ⊥ Departamento de Química, Facultad de Ciencias, Universidad del Bio-Bio, Concepción 405138, Chile; # Departamento de Química, Facultad de Ciencias, Universidad de Chile, Las Palmeras 3425, Ñuñoa, Región Metropolitana 7800003, Chile; ∇ Center of Materials and Nanotechnologies, Faculty of Chemical Technology, 48252 University of Pardubice, Nam. Cs. Legii 565, 53002 Pardubice, Czech Republic; ○ Central European Institute of Technology, Brno University of Technology, Purkyňova 123, 61200 Brno, Czech Republic; ◆ Institute of Advanced Materials (INAM), Universitat Jaume I (UJI), Avenida de Vicent Sos Baynat, s/n, Castellón de la Plana, 12071 Castellón, Spain; ¶ Instituto de Ciencia de los Materiales, Universidad de Valencia, C. José Beltrán 2, 46980 Paterna, Spain

**Keywords:** Ca−I complex, perovskite nanocrystals, anion exchange, surface passivation, Ca^2+^ doping

## Abstract

Anion exchange has been recognized as one of the most effective approaches for mediating the spontaneous formation of mixed-halide perovskite nanocrystals (MHPs) with tunable optical properties and color quality. However, the difference in the diffusion capability of halides, specifically between bromide and iodide species, into MHPs makes these materials prone to halide deficiency, which deteriorates their structural integrity and stability. In this work, we studied the surface passivation and composition engineering by introducing a dinuclear calcium-iodide scorpionate complex (CaI_SC_) dispersed into different organic solvents such as chloroform, dichloromethane, 1,2-dichloroethane, and acetonitrile, which favor or hinder the I-for-Br exchange process between this ligand and native CsPbBr_3_ perovskite nanocrystals (PNCs). By analyzing the CaI_SC_ content and the nature of the solvent, we are able to efficiently promote halide exchange, also generating an intermediate mononuclear Ca^2+^ complex, favoring Ca^2+^ doping and the diffusion of a high density of iodide species for triggering Pb^2+^ and halide defect compensation. From this strategy, suitable CaI_SC_-capped CsPbBr_3–*x*_I_*x*_ PNCs active layers were prepared for the fabrication of efficient down-light converters, with operational stability up to 480 h. This contribution offers an alternative for the processing of stable multicolor PNCs with facile modulation of their photophysical properties, making them adequate for the fabrication of future LED technologies.

## Graphical abstract

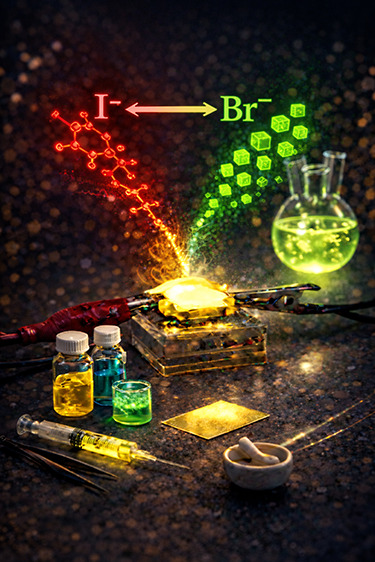

## 1 Introduction

Halide perovskite nanocrystals (PNCs) with APbX_3_ stoichiometry (where A = Cs^+^, formamidinium, FA^+^; X = Cl^–^, Br^–^, I^–^) are considered some of the most promising photoactive materials, mainly applied in the fields of optoelectronics and photovoltaics.^1,2^ This is due to their outstanding properties, such as a soft ionic lattice,^3^ versatile surface chemistry,^4^ and low-cost solution synthesis,^5^ as well as enhanced interparticle charge transport, high absorption coefficient,^6^ and pure color emissions with high photoluminescence quantum yield.^7^ These characteristics have led to significant breakthroughs in the fabrication of efficient light-emitting diodes (LEDs) with external quantum efficiencies up to 30% and good color quality,^8,9^ positioning them as competitive luminescent materials for next-generation liquid crystal display technologies. Through composition and ligand engineering, the halide-exchange process can be utilized to synthesize multicolor PNCs whose absorption and photoluminescence (PL) emission span the entire UV–visible-NIR spectrum.^10–13^ Given the similar ionic radii of halides, various mixed compositions can be obtained by combining pristine APbX_3_ (e.g., APbCl_3_ + APbBr_3_ or APbBr_3_ + APbI_3_), producing mixed-halide APbCl_3–*x*_Br_*x*_ and APbBr_3–*x*_I_*x*_ nanostructures with tunable optical properties,^14–16^ being an interesting alternative, for instance, in the spectroscopical detection of halides in aqueous solutions.^17^ However, due to the larger radii of halides when advancing in the group, the energy of Pb–X bonds decreases (Pb–Cl > Pb–Br > Pb–I),^18^ making the photoactive material prone to generate surface defect sites,^19^ inducing the eventual deterioration of the perovskite structural integrity and the quenching of its PL properties. Furthermore, considering the lability of halides in APbBr_3–*x*_I_*x*_ PNCs,^20,21^ some works highlight the appearance of phase segregation under illumination,^22,23^ producing Br/I-rich domains in the grain boundaries and Br/I-deficient domains in the center, thus splitting the PL emission of mixed-halide PNCs. In this context, iodide vacancies are formed, acting as primary nonradiative recombination centers,^24^ which is the main reason to decrease the optical performance in LEDs.^25^

To inhibit the formation of surface defects during anion exchange and thereby obtain mixed-halide perovskites (MHPs) with enhanced and stable PL, several strategies have been developed. These include the direct synthesis of CsPbBr_3–*x*_I_*x*_ PNCs with precise control over Br/I surface stoichiometry,^26^ surface passivation using alkyl/arylammonium halide salts with better binding capability than the conventional oleic acid (OA) and oleylamine (OLA) ligands,^27–29^ treatment with ascorbic acid where the elimination of deep trap states is maximized to avoid iodide release and material deterioration,^30^ and addition of metal halide salts^31,32^ and additives such as PbSO_4_ ^33^ and BF_4_^–^.^34^ These species suppress carrier energy traps and improve the radiative recombination pathway in PNCs. In a previous contribution,^35^ we have shown how the nature of ammonium halide ligands used for CsPbBr_3_ PNC stabilization in alcohol environments influences anion-exchange reactions with potassium iodide (KI), using two kinds of ammonium halide ligands: didodecyldimethylammonium bromide (DDAB) and benzyldodecyldimethylammonium bromide (BDAB). In the presence of DDAB, most of the Cs^+^ and Br^–^ deficiencies are compensated by this ligand, finding a low density of available halide vacancies to be filled by iodide species,^36^ generating white-emitting MHPs. Conversely, by adding BDAB to the perovskite dispersion, steric hindrance is induced in the material surface, passivating the structural defects partially and favoring the mobility of more iodide anions to occupy halide positions. Here, red-emitting PNCs are observed with a higher iodide content in the resulting photomaterial.

An interesting approach has also been addressed by Qi and co-workers,^37^ who have mediated the halide-exchange process in solid-state CsPbBr_3_ thin films by adding phenylphosphonic halide (PhPOX_2_, X = Cl^–^, I^–^) using hydrofluoroethers as solvents, covering the RGB color spectrum. In that work, the PhPOX_2_ can be hydrolyzed to release phenylphosphonic acid (PPOA) and their corresponding hydrohalic acids (HX), being these last species responsible to mediate the halide substitution on the CsPbBr_3_ surface. Simultaneously, PPOA is linked to the perovskite terminals through P=O···Pb coordination bonds and O–H···X hydrogen bonds, protecting the entire photoactive material. On the other hand, halide exchange has also been facilitated by triggering the photocatalytic oxidation of organic solvents such as chloroform (CHCl_3_, CHL) and dichloromethane (CH_2_Cl_2_, DCM), using CsPbBr_3_ PNCs as the photocatalyst. At this point, Sandeep and co-workers^38,39^ have reported the formation of alkyl radicals and the release of Cl^–^ anions, reaching the PNC surface for promoting Cl-for-Br substitution and generating a blueshift in the PL emission. In this context, it seems that the nature of the solvent also plays a pivotal role on the optical properties of the resulting MHPs; however, its impact on favoring/restraining the anion exchange has not been widely explored.

In this work, we studied the interaction and effect of a prominent capping ligand based on a dinuclear Ca–I scorpionate complex, denominated as bis­[(tetrahydrofuran)­(iodide)­(μ-κ^3^-2,2-bis­(3,5-dimethylpyrazol-1-yl)-1,1-bis­(4-(diethylamino)­phenyl)­ethanolate)­calcium­(II)] or [CaI­(κ^3^-bpzbdeape)­(μ-O)­(thf)]_2_ (CaI_SC_) (see Scheme S1 in the Supporting Information), on the photophysical properties of pristine CsPbBr_3_ PNCs. CaI_SC_ has been recently used in organic chemistry applications for catalyzing the copolymerization of epoxides and cyclic anhydrides.^40^ By dispersing the CaI_SC_ ligand in several types of organic solvents, such as CHL, DCM, 1,2-dichloroethane (C_2_H_4_Cl_2_, DCE), and acetonitrile (CH_3_CN, ACN), in the presence of pristine CsPbBr_3_ PNCs, it is possible to trigger the fragmentation of its dinuclear Ca^2+^ structure by breaking the Ca−μ–O–Ca connectivity. This fact induces partial Ca^2+^ doping and oxygen incorporation into the perovskite, passivating Pb^2+^ and halide defects during the I-for-Br exchange. Accordingly, we were able to prepare suitable CaI_SC_ inks for the fabrication of luminescent color converter LEDs with a long-term stability of up to 480 h under continuous operation. This approach provides a promising scenario for the fabrication of stable multicolor MHP active layers for the development of efficient optoelectronic devices.

## 2 Experimental Section

### 2.1 Synthesis of CsPbBr_3_ PNCs

CsPbBr_3_ PNCs were synthesized with some modifications by a hot-injection method using Cs-oleate and PbBr_2_ solutions in stoichiometric amounts. First, a Cs-oleate solution was prepared by mixing 0.407 g of Cs_2_CO_3_ (202126, 99.9%, Sigma-Aldrich), 1.4 mL of OA (364525, 90%, Sigma-Aldrich), and 20 mL of 1-octadecene (1-ODE, O806, 90%, Sigma-Aldrich) in a 50 mL three-necked flask, under vigorous stirring at 80 °C and vacuum for 30 min. Then, the temperature was increased to 120 °C and the mixture was kept under vacuum for 30 min. The mixture was heated to 150 °C under a N_2_ atmosphere. The resulting transparent solution was maintained at 120 °C for further use. In a separate 100 mL three-necked flask, 0.9 g of PbBr_2_ (ABCR; AB202085, 99.998%) was mixed with 50 mL of 1-ODE. The mixture was heated at 120 °C under vacuum with vigorous stirring for 1 h. Subsequently, a preheated mixture of 5.0 mL each of OA and OLA (HT OA100, 98%, Sigma-Aldrich) was added to the reaction flask to promote PbBr_2_ dissolution. The temperature of the PbBr_2_ mixture was rapidly increased to 180 °C, at which point 4 mL of preheated Cs-oleate was swiftly added. After injection, a green precipitate was obtained for the CsPbBr_3_ colloidal solution. The reaction flask was quickly immersed in an ice–water bath for 5 s to quench the reaction.

The PNCs were isolated and purified by using a two-step washing procedure. First, the crude solution was mixed with methyl acetate (MeOAc, 296996, 99.5%, Sigma-Aldrich) in a 1:2 volume ratio (e.g., 30 mL of crude solution with 60 mL of MeOAc) and centrifuged at 5000 rpm for 5 min. The supernatant was discarded, and the pellet was redispersed in 5 mL of hexane (CHROMASOLV, 34859, 99.7%, Honeywell). This dispersion was then mixed with 10 mL of MeOAc and centrifuged again under the same conditions. After discarding the supernatant, the final purified PNC pellet was redispersed in hexane to a concentration of approximately 100 mg·mL^–1^ for storage and further characterization.

### 2.2 Synthesis of [CaI­(κ^3^-bpzbdeape)­(μ-O)­(thf)]_2_ (CaI_SC_)

The synthesis of the calcium complex, denoted as CaI_SC_, proceeds via the preparation of two key precursors: bis­(3,5-dimethyl-1H-pyrazol-1-yl)­methane (bdmpzm) and the ligand 2,2-bis­(3,5-dimethyl-1H-pyrazol-1-yl)-1,1-bis­(4-(diethylamino)­phenyl)­ethanol (bpzbdeapeH). The synthetic protocols for these compounds are adapted from established literature and are summarized below.^40^

### 2.3 Preparation of Bis­(3,5-dimethyl-1H-pyrazol-1-yl)­methane (bdmpzm)

A mixture of 3,5-dimethylpyrazole (5.00 g, 52.00 mmol), tetrabutylammonium bromide (0.80 g, 2.50 mmol), anhydrous K_2_CO_3_ (7.10 g, 52.00 mmol), and powdered KOH (3.10 g, 55.00 mmol) in CH_2_Cl_2_ (200 mL) was refluxed for 8 h. The reaction mixture was filtered, and the organic phase was dried over anhydrous MgSO_4_. After filtration and concentration under reduced pressure, the crude solid was treated with powdered KOH (3.00 g) for 2 h. The product was then extracted with diethyl ether, filtered, and concentrated. The product was obtained as a white, crystalline solid in 86% yield.

### 2.4 Preparation of 2,2-Bis­(3,5-dimethyl-1H-pyrazol-1-yl)-1,1-bis­(4-(diethylamino)­phenyl)­ethanol (bpzbdeapeH)

To a solution of bdmpzm (1.00 g, 4.89 mmol), 3.06 mL (4.89 mmol) of *n*-butyllithium (*n*-BuLi, 1.6 M in hexane), 1.03 g (4.89 mmol) of 4,4′-diethylbenzophenone, and 70 mL of tetrahydrofuran (THF) were mixed, generating a white precipitate (bpzbdeapeH ligand). The solid was washed with 50 mL of hexane for 18 h and dried under vacuum, producing a crystalline powder with 88% yield. Last, for the synthesis of the Ca complex, bpzdeapeH ligand (0.50 g, 0.94 mmol) was dissolved in dry THF (20 mL) and cooled to −78 °C. Then, *n*-BuLi (1.6 M in hexane, 0.58 mL, 0.94 mmol) was added dropwise, and the mixture was kept at −78 °C for 1 h. The resulting solution was transferred to a precooled suspension of CaI_2_ (0.28 g, 0.94 mmol) in THF (20 mL). After heating to room temperature and stirring overnight, the solvent was removed under vacuum and the residue was washed twice with *n*-hexane, producing a white solid with 75% yield.

### 2.5 Preparation of CaI_SC_-Capped CsPbBr_3–*x*_I_*x*_ Nanocrystals in Organic Solvents

A diluted dispersion of CsPbBr_3_ PNCs was prepared at a concentration of 10 mg·mL^–1^ from a 100 mg·mL^–1^ stock solution in hexane. Then, four different stock solutions of the capping agent CaI_SC_ were prepared by dissolving 36 mg of the complex in 1200 μL of the following solvents: CHL, DCM, DCE, and ACN, generating a stock solution of 30 mg·mL^–1^. To study the capping process, 2.4 mL of the diluted perovskite dispersion was added to four separate vials, and later, 0, 100, 200, 300, and 400 μL of the CaI_SC_ solution was added to the respective vials; to ensure identical dilution conditions in all samples, 400, 300, 200, 100, and 0 μL of the corresponding organic solvent was added, respectively, achieving a total volume of 400 μL.

### 2.6 Characterization of Morphology, Structure, Optical Properties, and Surface Environment of the CaI_SC_-Capped CsPbBr_3–*x*_I_*x*_ Nanocrystals

The morphology of CaI_SC_-capped CsPbBr_3–*x*_I_*x*_ PNCs was analyzed through high-resolution transmission electron microscopy (TEM) using a field emission transmission electron microscope (Hitachi HF-3300, with a bias acceleration voltage of 300 kV). The average particle size of PNCs was obtained from the TEM images using ImageJ software. The crystalline phase of PNCs was analyzed through selective area electron diffraction (SAED) patterns and from the corresponding X-ray diffraction (XRD) profiles. A D4 Endeavor diffractometer from Bruker-AXS was used, using a Cu Kα radiation source (λ = 1.54056 Å) with the following factors: 2θ range of 5°–80° (0.02°/step and 1.2 s/step). UV–vis spectra of films were acquired by using a UV–vis absorption spectrophotometer (JASCO V-730) in the wavelength range of 375–750 nm, while steady-state and time-resolved PL measurements were conducted through a PL spectrophotometer (JASCO FP-6200). An excitation wavelength of 420 nm was used to perform the steady-state PL measurement. Surface chemical composition and electronic state of PNCs were determined by X-ray photoelectron spectroscopy (XPS, ESCA-2SR, Scienta-Omicron). Spectra were recorded using monochromatic Al Kα = 1486.6 eV. The following sequences of spectra were recorded: survey spectra, C 1s, Cs 3d, Ca 2p, Pb 4f, Br 3d, I 3d, O 1s, N 1s, and C 1s again to verify the stability of the charge as a function of time. The survey and high-resolution spectra were recorded at a pass energy of 150 and 20 eV, respectively. The binding energy scale was referenced to adventitious carbon (284.8 eV). CasaXPS processing software (Casa Software Ltd.) was used to analyze the data, and the quantitative analysis was made using sensitivity factors provided by the manufacturer. NMR spectra were recorded on a Bruker Ascend TM-500 spectrometer and referenced to the residual deuterated solvent, operating at 500.16 MHz for ^1^H nuclei. The analyses in the above-mentioned deuterated solvents were carried out using 1.0 s of relaxation delay, a 30° pulse of 3.33 μs, 16 scans, and a gain of 64. The FID was acquired into 64 K data points during a 3.2768 s acquisition time, and the data were processed with 0.30 Hz line broadening.

### 2.7 Fabrication of Down-Light Converting Devices

For the fabrication of down-light converters using CaI_SC_-capped CsPbBr_3–*x*_I_*x*_, first, the perovskite nanoparticle inks prepared with CHL, DCM, DCE, and ACN (perovskite:CaI_SC_ dispersion volume ratio = 12.5:1) were mixed with a commercial acrylate-based resin (GEMA LABORATORIES, UV) in a perovskite:resin volume ratio = 1:2, stirred vigorously for 30 min, and left to stand for 24 h. All composites were deposited onto a blue LED chip (3 W and wavelength of 445–450 nm). By applying a constant voltage of 7.0 V for 5 min, the monomer curing was carried out, obtaining the polymeric active layer. The stability measurements of the light converters were performed by studying their respective PL emission spectrum in a photoluminescence spectrophotometer (JASCO FP-6200), varying the aging time at 7.5 V, reaching a maximum continuous operational time of 480 h.

### 2.8 Computational Details

The electronic structure for all periodic perovskites (CsPbBr_3_ and CsPbI_3_) here built were fully computed by using FHI-aims software.^41,42^ PBE was selected as the DFT xc-functional along with tight basis set settings including relativistic (ZORA) and spin–orbit coupling (SOC) effects. A k-grid of 12 × 8 × 12 was employed to describe each perovskite. The optimization was carried out by using the BFGS optimizer with an optimization threshold of 0.005 eV Å^–1^. We also considered the VdW correction in our calculation. For the nonperiodic molecular systems, the ORCA quantum chemistry package (version 6.1.1) was employed.^43,44^ The electronic structure of the selected molecular models was described by the r^2^-SCAN-3c composite^45^ along with the SMD implicit solvent model to describe the solvent effect.^46^ The Minimal Basis Iterative Stockholder (MBIS)^47^ charge analysis was also computed on the optimized structures. Avogadro2 ^48^ and ChimeraX^49^ were extensively used to visualize and plot the partial atomic charges and electrostatic potential maps.

## 3 Results and Discussion

With the purpose of promoting the anion exchange in green-emitting CsPbBr_3_ PNCs, CaI_SC_ was employed as the iodide source. This strategy has been proposed to enhance the carrier transport and surface defects passivation in semiconductor nanoparticles.^50^ CaI_SC_ stock solutions were prepared in different solvents to assess their effects on the exchange process: CHL, DCM, DCE, and ACN at 30 mg·mL^–1^. Different volumes of the respective CaI_SC_ stock solutions (0–400 μL) were added to the CsPbBr_3_ PNC colloidal solutions, but a total volume of 2.8 mL was kept to suppress dilution effects. See Supporting Information (SI) for details. Figure 1A shows how the color of the halide perovskite emission dramatically changes after the addition of CaI_SC_ in chlorinated solvents. This is a clear indication that anion exchange occurs. On the contrary, the color barely changed when the CaI_SC_ complex was dissolved in ACN. This reveals a clear effect of the solvent. TEM images of PNC samples in the absence and presence of CaI_SC_ show the typical nanocube-type morphology (see Figures 1B–E, B′–D′, and S1) with an average particle size between ∼ 8 and 11 nm. However, before adding the Ca–I complex, we noted that the nature of the organic solvent influences the nanoparticle size. In this context, the higher the solvent polarity (ACN > DCM > CHL > DCE), the bigger the average size; see Figure S2A–D. The use of a polar solvent can induce the partial removal of OA and OLA ligands linked to PNCs (in the form of oleylammonium oleate), favoring the emergence of structural defects and subsequent material destabilization.^51^ Therefore, the coalescence of PNCs is promoted to compensate for the defect sites, decreasing the surface energy.^52^ Then, by adding a progressive content of CaI_SC_ into the colloidal PNCs, in the presence of the chlorinated solvents, the nanocube shape is maintained (also for the 200 μL case, see Figure S1A–C), producing a bigger particle size; see Figure S2A′–C′,A″–C″. This is a clear indication of the I-for-Br exchange produced between CaI_SC_ and CsPbBr_3_ PNCs, making it possible for a higher density of available iodide to diffuse to the perovskite lattice, occupying halide positions. On the contrary, halide substitution was not induced in the presence of ACN, presenting nanocubes with a similar particle size (see Figure S2D′,D″) coexisting with different shapes such as nanospheres and nanowires; see Figures 1E′ and S1D. We suggest that this solvent interacts directly with the nanoparticle surface, restraining the iodide migration to reach the perovskite and deteriorating its structural integrity.

**Figure 1:**
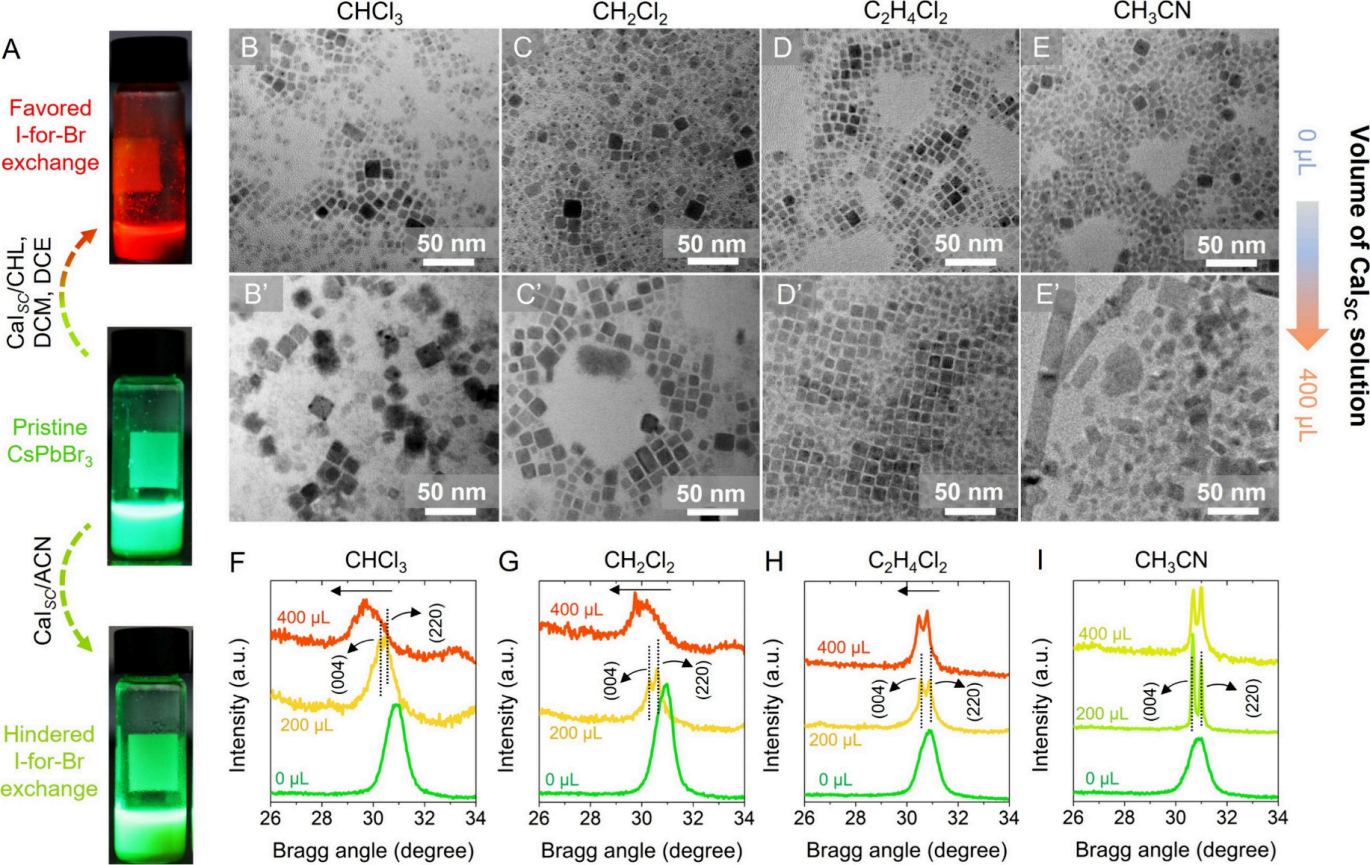
(A) Pictures of the suspension of CsPbBr_3_ PNCs before (center) and after anion exchange with CaI_SC_ in chlorinated solvents (on top, CHL, DCM, DCE) and nonchlorinated solvent (ACN). TEM images of PNCs after adding different volumes of CaI_SC_ stock solution: (B, C, D, E) 0 μL and (B′, C′, D′, E′) 400 μL in the presence of (B, B′) CHL, (C, C′) DCM, (D, D′) DCE, and (E, E′) ACN. (F–I) Low-angle XRD peaks of (004) and (220) planes of PNCs before and after promoting halide exchange with different volumes of the CaI_SC_ ligand in the presence of (F) CHL, (G) DCM, (H) DCE, and (I) ACN.

To get preliminary insight into the crystalline structure of CsPbBr_3–*x*_I_*x*_ MHPs after the addition of CaI_SC_, SAED patterns were obtained. As seen in Figure S3, we identified the presence of the orthorhombic phase (ICSD 97851)^53^ independent of the organic solvent used for the Ca-complex dissolution. Then, XRD measurements were conducted to analyze the modification of the perovskite lattice after iodide incorporation during halide substitution. As seen in Figure S4, typical XRD peaks are associated with the (110), (111), (220), (130), (312), and (224) planes of the orthorhombic phase of CsPbBr_3_, confirming the crystalline phase noted by SAED. Furthermore, by inducing the I-for-Br exchange after adding the CaI_SC_ ligand in the presence of chlorinated solvents, the XRD pattern of PNCs is shifted to lower Bragg angles, this shift being more evident as the Ca-complex content increases; see Figure 1F–I. This change is due to the enlargement of the [PbX_6_]^4–^ octahedra units^18^ as a consequence of the larger ionic radius of iodide compared with bromide species. To support this statement, a set of electronic structure calculations based on density functional theory (DFT) was carried out by building two representative models of CsPbBr_3_ and CsPbI_3_ PNCs which were subsequently optimized at the PBE level in periodic boundary conditions^42^ (see computational details); see Figure S5. The computed Pb–Br and Pb–I bond lengths were ∼ 3.03 and ∼ 3.24 Å, respectively. Conversely, no displacement in the XRD pattern was observed in the PNC suspension with ACN. This result confirms the restrained anion-exchange process between the PNCs and CaI_SC_. Accordingly, the use of chlorinated solvents favors efficient halide substitution, preserving the crystalline phase and integrity of PNCs unlike the addition of ACN, where the halide diffusion is hindered. Last, the addition of the CaI_SC_ complex allows us to detail the splitting of the (004) and (220) planes, indicating the emergence of the octahedra tilting during anion exchange.

The photophysical properties of CsPbBr_3_ PNCs during the halide-exchange process were studied to investigate the dynamics of the process. Absorption and steady-state PL spectra of PNC samples are shown in Figures 2A–C and S6A–C. A redshift (from green → red) in the absorption edge and the emission peak position are detected in the samples with chlorinated solvents; see Table S1. Taking into account that the typical absorption edge and PL emission of pure CsPbI_3_ PNCs appear at 647 and 678 nm,^18^ respectively, we deduce that iodide species from CaI_SC_ partially fill/replace bromide positions into CsPbBr_3_ PNCs, producing CsPbBr_3–*x*_I_*x*_ MHPs. However, depending on the nature of the chlorinated solvent, the iodide incorporation dynamics can be accelerated or delayed, requiring a higher or lower CaI_SC_ content for producing the corresponding red-emitting PNCs. At this point, time-dependent PL measurements revealed different dynamics of anion exchange. As seen in Figures 2A′–C′ and S7A–C, the redshift in the PL of MHPs occurs faster in the presence of DCM, followed by CHL, while anion exchange is delayed in the DCE medium (1.23, 1.82, and 2.66 min^–1^, respectively). As we highlighted above, the use of polar solvents shows a strong impact on the surface chemistry of PNCs, causing the detachment of a high content of OA/OLA ligands, which would leave highly Br-deficient PNCs to be compensated through iodide incorporation. Therefore, a solid solution can form more rapidly with a low CaI_SC_ concentration. On the contrary, the low polarity of DCE provides a low density of defect sites in PNCs, taking more time for halide substitution. Conversely, in the case of ACN, it is confirmed that halide substitution is restrained, exhibiting no considerable changes in the optical properties of PNCs; see Figures 2D,D′ and S6D. Although ACN shows a higher dipole moment compared with DCM (3.4 vs. 1.6), we suggest that the former solvent interacts with the PNCs, hindering the I-for-Br exchange with the CaI_SC_ complex.

**Figure 2:**
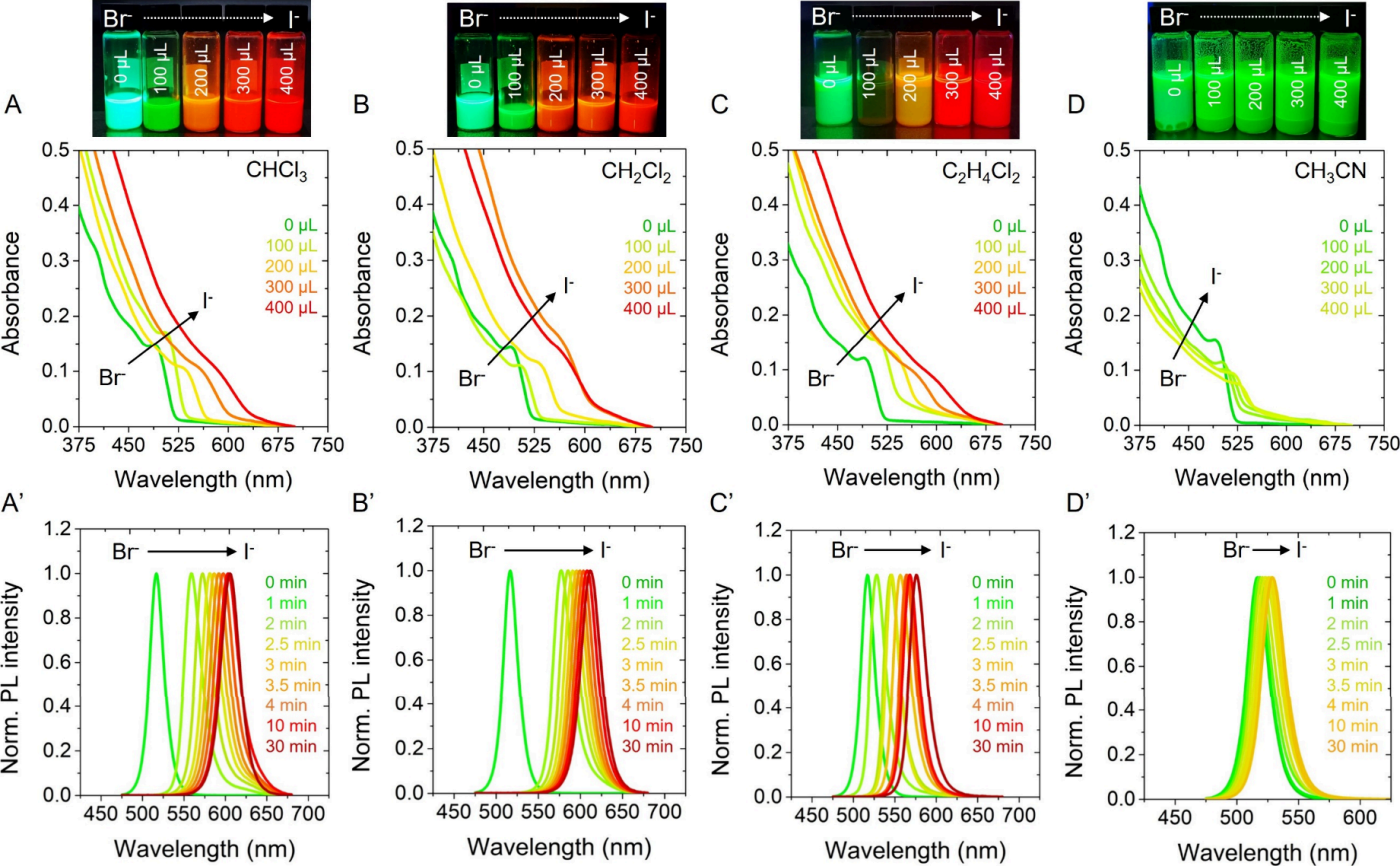
(A–D) UV–vis absorption and (A′–D′) normalized time-dependent PL emission spectra of CsPbBr_3_ PNCs before and after adding different volumes of CaI_SC_ ligands in (A, A′) CHL, (B, B′) DCM, (C, C′) DCE, and (D, D′) ACN. Photographs on top of the figure show the evolution of the emission’s color (from green to red) in the PNCs upon anion exchange in the different organic solvents.

Last, stability of PNCs in the organic solvents was evaluated through PL measurements over time for two types of scenarios: (i) in the absence of CaI_SC_ ligand and (ii) after adding 300 μL of CaI_SC_ dispersed into the organic environments (see Figure S8). Under the influence of chlorinated media, the PL intensity of CsPbBr_3_ PNCs increases, deducing a progressive compensation of defect sites on the material surface. Without the CaI_SC_-mediated post-treatment of PNCs, we suggest the incorporation of atmospheric oxygen to passivate partially the halide deficiency in the perovskite, improving the radiative recombination pathway.^54^ Later, after promoting the iodide-for-bromide exchange with the Ca–I complex, we observed a progressive redshift in the PL peak position along time (from 0 to 864 h) with an increase in the emission intensity. We hypothesize that the higher the solvent polarity (DCM and CHL), the stronger the partial removal of surface oleate and oleylammonium anions (linked to Cs^+^ and Br^–^ site species, respectively),^55^ showing that more iodide can diffuse rapidly toward the perovskite lattice to produce the mixed halide. However, after 1512 h of stability in these solvents, the PL intensity begins to decrease, indicating material degradation, possibly due to the loss of labile iodide. Conversely, the emergence of the resultant PL feature in the solid solution into DCE is slower, and a subsequent increase in the PL intensity compared with the two previous cases is associated with a more delayed halide substitution. In the case of ACN, the PL intensity quenching of the nonmodified PNCs is accelerated as a result of the high volume of the polar solvent, favoring the structural deterioration. Accordingly, we conclude that it is possible to modulate the rate of the halide-exchange phenomenon as a function of the solvent polarity, offering a prominent alternative for preparing CsPbBr_3–*x*_I_*x*_ PNCs with a tunable band gap.

The impact of halide exchange on the surface chemistry of the resulting MHPs was further analyzed through XPS measurements. Through the survey spectra (see Figure S9), the coexistence of C, O, N, Pb, Cs, Ca, Br, and I in the perovskite samples was identified, summarizing their chemical composition in Tables S2 and S3. In this way, high-resolution (HR) XPS spectra of the main elements were acquired in order to obtain information about their chemical speciation. Figure S10A_1_–A_4_ shows the HR-XPS Cs 3d spectra of CaI_SC_-capped PNCs, evidencing a doublet at 724/738 eV due to SOC. These signals are ascribed to the Cs^+^ cations contained in the A-positions of the CsPbX_3_ lattice.^56^ Then, HR-XPS Pb 4f spectra depict the 4f_7/2_/4f_5/2_ core levels at ∼ 138/143 eV, characteristic of Pb^2+^ species conforming the [PbX_6_]^4–^ building blocks; see Figure S10B_1_–B_4_. Then, the formation of Pb–Br and Pb–I bonds into the perovskite structure is confirmed through the HR-XPS Br 3d and I 3d spectra, exhibiting their corresponding 3d_5/2_ and 3d_3/2_ doublets at 68/69 eV and 619/630 eV,^12^ respectively; see Figure S10C_1_–C_4_,D_1_–D_4_. HR-XPS N 1s spectra of PNC samples (see Figure S10E_1_–E_4_) show two main signals at 400 and 402 eV attributed to alkylamine (R-NH_2_) and alkylammonium species (R-NH_3_^+^) surrounding the PNC surface,^57^ respectively. These species are assigned to the presence of OLA and oleylammonium species (OLA^+^) surrounding the PNC surface. Last, HR-XPS Ca 2p spectra are also presented, emerging a 2p_3/2_/2p_1/2_ doublet at ∼ 348/351 eV^58^ associated with Ca^2+^ species from the CaI_SC_ complex (see Figure 3A). We hypothesize that the coexistence of amine species and Ca^2+^ cations is a strong indication of surface passivation provided by the Ca complex after halide substitution.

**Figure 3:**
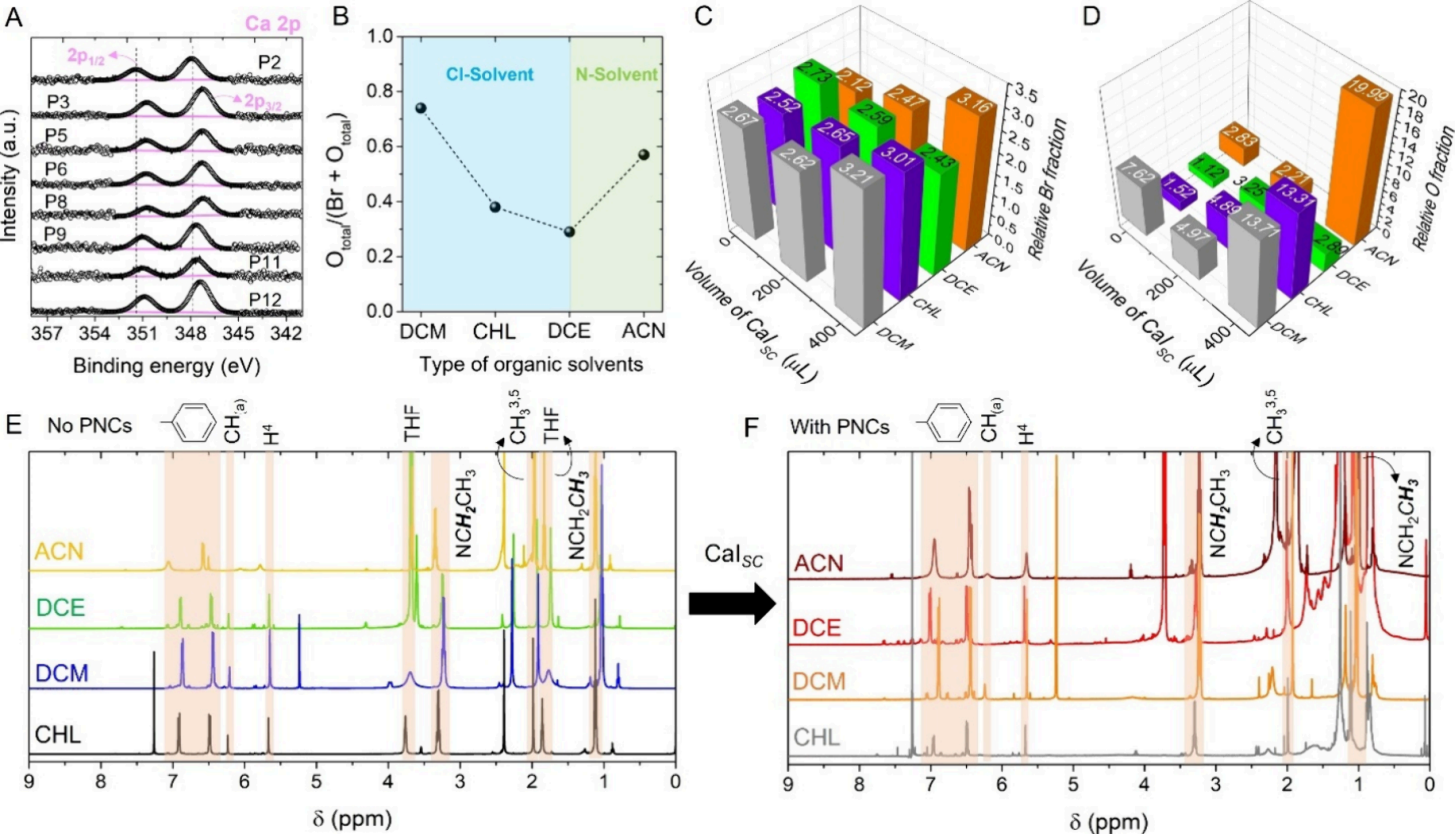
(A) HR-XPS Ca 2p spectra of CaI_SC_-capped CsPbBr_3–*x*_I_*x*_ PNCs after adding (P2) 200 and (P3) 400 μL of CaI_SC_ in CHL, (P5) 200 and (P6) 400 μL of CaI_SC_ in DCM, (P8) 200 and (P9) 400 μL of CaI_SC_ in DCE, and (P11) 200 and (P12) 400 μL of CaI_SC_ in ACN. (B) Total oxygen-to-bromide ratio [O_total_/(Br+O_total_)] estimated in CsPbBr_3_ PNCs before the addition of CaI_SC_ ligand in the presence of different organic solvents. Relative (C) bromide and (D) oxygen fractions of PNCs before and after addition of different volumes of the CaI_SC_ ligand as a function of the organic solvent nature. ^1^H (500.16 MHz, deuterated CHL, DCM, DCE, and ACN, 298 K) NMR spectra of the CaI_SC_ ligand in the (E) absence and (F) presence of CsPbBr_3_ PNCs, varying the nature of the organic solvent. Representative signals of the main organic functionalities of the Ca–I complex are highlighted in light orange color.

Going deeper into the XPS studies (see Table S3), it is worth noting that the oxygen content in PNCs [denoted as O_total_/(Br + O_total_) ratio] is increased in parallel with the polarity of the respective organic solvent, keeping the behavior as DCM > CHL > DCE for the chlorinated environments; see Figure 3B. This can corroborate the partial passivation of halide defects promoted by oxygen incorporation, thereby enhancing the optical properties of PNCs. On the contrary, although the O fraction by using ACN is high, this value is lower compared to employing lesser polar DCM, inferring that ACN molecules also play an important role in stripping surface oleate species from CsPbBr_3_ PNCs. Simultaneously, R-NH_2_ and R-NH_3_^+^ species increase gradually in the perovskite samples by raising the amount of CaI_SC_ in the chlorinated solvents. We ascribed this trend to the partial ligand exchange in between Ca–I complex (through the amine group coming from the 4-((diethylamino)­phenyl)­ethanolate moieties and oleylammonium cations), promoting the release of these native ligands in the form of OLA (formed by acid–base reaction in the presence of free OA),^59^ also compensating Cs^+^ defect sites. Surprisingly, the higher the CaI_SC_ content, the higher the relative Br^–^ and O fractions (see Figure 3C,D), suggesting that bromide species are more exposed in the material surface, while oxygen from the internal Ca–O bonds contained in the Ca–I complex also contributes to passive halide vacancies. This fact allows us to determine the possible breaking of the dinuclear Ca–I complex structure to produce mononuclear surface-bound mononuclear units. Last, a higher Ca^2+^ fraction is achieved in the presence of more CaI_SC_, also compensating Br^–^ vacancies via iodide incorporation [denoted as I/(Br + I) ratio]. At this point, we suggest the introduction of Ca^2+^ from the mononuclear Ca–I units to substitute/fill undercoordinated Pb^2+^ species into the perovskite lattice. This is deductible since the binding energy of Pb 4f doublets is shifted toward lower values (∼ 0.1–0.2 eV), considering the lower electronegativity of Ca^2+^ compared with Pb^2+^. Accordingly, we propose the Ca^2+^doping of CsPbBr_3–*x*_I_*x*_ PNCs, allowing us to affirm that the use of a Ca-halide complex would favor the surface passivation of Schottky defects (in order to maintain the electroneutrality of the perovskite structure), which can amplify their photophysical properties and stability for optoelectronic devices.

To get a closer inspection of the CaI_SC_ and CsPbBr_3_ PNC interaction under the influence of different organic solvents, nuclear magnetic resonance (NMR) measurements were performed. This spectroscopic analysis aimed to identify the NMR signals of CaI_SC_ in the absence and presence of PNCs (see Figure 3E,F), respectively, demonstrating some modifications in the main structure of the complex shown in Scheme S1, verifying the stability of the Ca compound in the presence of PNCs and corroborating its interactions with the perovskite material. The ^1^H NMR spectra of the CaI_SC_ compound in different deuterated solvents show the resonances corresponding to protons of the scorpionate ligand and the THF molecule coordinated to the Ca atom (see Figures S11A–S14A). Analysis of the ^1^H NMR spectra of the isolated solid after adding the CaI_SC_ complex to CsPbBr_3_ PNCs (see Figures S11B–S14B) shows the resonances corresponding to protons of the Ca–I scorpionate ligand coordinated to the Ca atom without any decomposition resonances or resonances of the free ligand being observed. To support this point, a molecular model of CaI_SC_ structure was built and optimized at the DFT r^2^-SCAN-3c level^45^ in SMD implicit solvent;^44,46^ see Figure S15. The optimized structure shows that the whole dinuclear–Ca-I complex structure was fully kept for all considered solvents (CHL, DCM, DCE, and ACN), showing its inherent stability in organic media with different polarities in the absence of PNCs. It should be noted that in all solvents, the signals corresponding to the THF ligand have disappeared, which indicates the presence of CaI_SC_-PNC interactions. It is important to highlight that the resonances assigned to methyl groups of pyrazole rings (∼ 1.80 to 2.60 ppm) appear broad compared to those observed in the spectra where only the CaI_SC_ complex is present. These facts could corroborate the coordination of the compound to the perovskite nanoparticle structure, indicating that the Ca–O–THF bond and the Ca−μ–O–Ca connectivity are broken likely due to the direct Ca^2+^ and Br^–^ interaction in the perovskite surface. In fact, a bond analysis study based on DFT through the Pipek–Mezey (PM) localization method^44^ shows that the Ca­(II) metallic center only forms bonding overlap with iodine and with both oxygen atoms present on the Ca−μ–O–Ca bridge associated with the scorpionate ligand; see Figure S16. The calcium bimetallic structure is therefore probably maintained through the Ca−μ–O–Ca bridge of scorpion ligands through electrostatic interaction. The bond analysis also shows that THF-Ca­(II)-complex interaction is weaker, or at least no bonding overlap was detected which corroborate the lability found experimentally.

Under this premise, we propose the main Ca–O coordination bonds of the CaI_SC_ fragment upon contact with the CsPbBr_3_ PNCs, deducing the following mechanism: Considering that Pb^2+^ from the PNC surface exhibit a stronger Lewis acid character than Ca^2+^, the μ-O can coordinate to Pb^2+^ sites, thereby polarizing the Ca–O bond, weakening it, and facilitating the cleavage of the Ca−μ–O–Ca bridges. Subsequently, Ca^2+^ cations released from the fragmented CaI_SC_ ligand become anchored to Br^–^ species on the PNCs (two Br^–^ anions can compensate both the oxidation state and the covalent valence of one Ca^2+^). This interaction favors iodide release, mediating the anion exchange and the subsequent breaking of the Ca-THF interactions; see Scheme 1. At this point, the surface-mediated monomerization of the CaI_SC_ is triggered, where the [CaI­(κ^3^-bpzbdeape)­(μ-O)­(thf)]_2_ dinuclear structure splits into mononuclear units. Meanwhile, the bulky bpzbdeape ligand ensures that the calcium center remains isolated as mononuclear sites on PNCs. Alternatively, the coordinated THF molecules are released, suggesting that less steric hindrance is produced by the monomeric units, allowing the metal center to bind to the perovskite surface. In this context, the Ca mononuclear units can introduce calcium and oxygen species, as evidenced by XPS analysis. Finally, iodide species from the mononuclear Ca sites will be available for halide exchange, favoring the iodide migration to compensate/substitute Br^–^ positions, producing a remarkable change in the optical features of the resulting photoactive material.

**Scheme 1:**
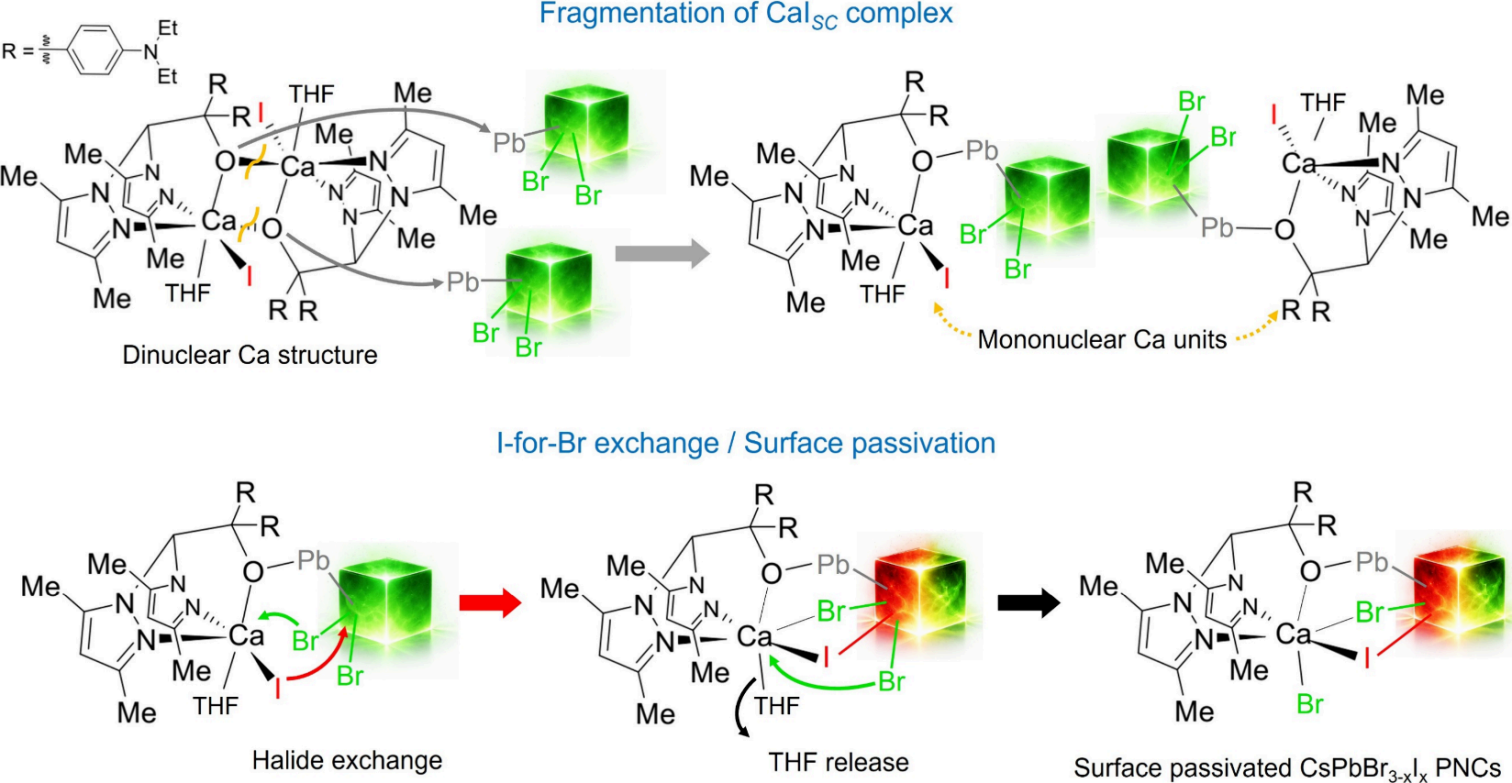
Representative Illustration of the CaI_SC_ Complex Cleavage and Subsequent Anion Exchange, Producing Surface-Passivated MHPs

After elucidating the influence of CaI_SC_ on both surface defect compensation and halide-exchange process in CsPbBr_3_ PNCs, we proceed to employ the as-prepared CaI_SC_-capped CsPbBr_3–*x*_I_*x*_ colloidal inks in the fabrication of down-light converters. For this aim, first, we have embedded the respective nanoparticle samples, dispersed in the studied organic solvents, into a commercial acrylate-based polymeric resin with a perovskite:resin volume ratio = 1:2, and later, deposited as active layers on a blue-emitting LED (3 W, 440–450 nm emission wavelength); see Supporting Information for further details. Then, we vary the operation time in the resulting devices by applying a constant voltage of 7.5 V, with the purpose of analyzing the retention of the initial PL intensity as a direct measure of the light converter stability. In the presence of chlorinated environments, several differences were found for CaI_SC_-capped PNC light-converting devices. By using CHL solvent during luminescent ink preparation (see Figure 4A), the highest PL intensity was achieved, which is in good agreement with the highest relative calcium and iodide contents estimated for this perovskite material, inferring the full compensation of Pb^2+^ and Br^–^ defects by CaI_SC_ during the anion-exchange process, favoring the radiative recombination pathway. Nevertheless, a fast PL quenching together with a blueshift in the emission peak position was noticeable with time, indicating that the active layer was deteriorated under continuous light exposure. This phenomenon arises from the photooxidation of the perovskite layer, which is associated with the high lability of iodide species.^20,60^ Because Pb–I bonds possess a lower complexation affinity than Pb–Br bonds,^22^ iodide ions can more readily migrate toward the material surface, where they may subsequently sublime as I_2_.^60^ As a result, the perovskite becomes progressively enriched with Br species. This process also promotes the formation of iodide vacancies, which act as primary nonradiative recombination centers and gradually quench the PL emission.^61^ Consequently, the PL blueshift observed in the light converters proceeds in a monotonic and irreversible manner over time.

**Figure 4:**
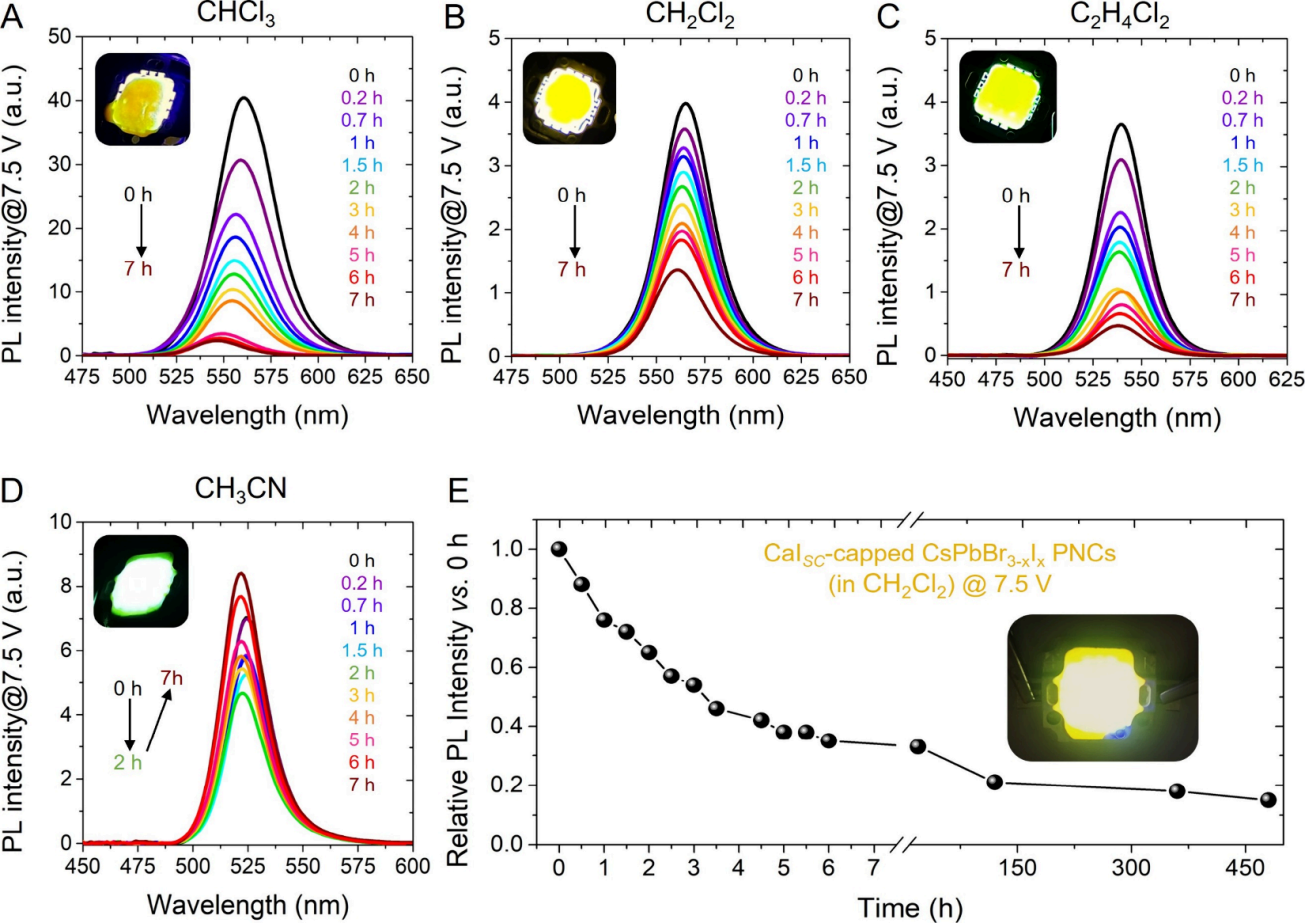
PL spectra of polymeric luminescent down-light converters using as-prepared CaI_SC_-capped CsPbBr_3–*x*_I_*x*_ PNCs inks in the presence of (A) CHL, (B) DCM, (C) DCE, and (D) ACN by applying a constant voltage of 7.5 V at different operational times. (E) Long-term stability of a CaI_SC_-capped CsPbBr_3–*x*_I_*x*_ (in DCM) light converter for 480 h under continuous operation.

Then, although the relative Ca^2+^ and I^–^ fractions estimated for PNCs by employing DCM are lower compared with the previous case, the highest oxygen fraction promoted by surface passivation of mononuclear Ca units [denoted as O_total_/(Ca + O_total_)] was achieved (see Table S3), allowing us to deduce that a better protective coverage was facilitated during the CaI_SC_ cleavage. This fact can explain the slow degradation of the PL emission of the light-converting device as a direct result of its enhanced operational stability after 7 h; see Figure 4B.

Finally, the light converter fabricated with the PNC active layer in the presence of DCE produces a lower PL intensity and operational resistance over time (see Figure 4C), ascribed to the detection of lower calcium, iodide, and oxygen fractions in the perovskite ink. Here, we deduce that both I-for-Br substitution and surface passivation via mononuclear Ca units are restrained, leaving the nanoparticle surface more exposed to ambient air and/or direct contact with acrylate polymer resin, causing progressive material deterioration. On the contrary to the above-mentioned results, the typical PL of the light-converting active PNC layer, prepared with ACN as the solvent at 525 nm, decreases after 2 h, but this signal is eventually increased after 7 h of continuous operation; see Figure 4D. Taking into account that halide substitution is negligible in this scenario, we associated the above trend to the surface modification via perovskite-ACN interaction, detaching some of the native surface ligands during the active layer preparation (solvent polarity), which is delayed in the presence of the polymeric resin. However, ACN, as a Lewis base, can coordinate with Pb^2+^ though using the lone pair of electrons on the nitrogen atom in its cyano group (−C≡N), suppressing the surface trap states.^62^ Therefore, a better PL stability in the light converter device is expected compared with the colloidal solution; see Figure S8D′. Electrostatic potential map computed at the DFT level for all assessed organic solvents shows that ACN locates a strong negative charge close to the nitrogen atom producing a high dipole moment with potential surface activity, which would produce the poisoning of the perovskite surface on different defect sites; see Figure S17.

Attending to the LED performance analyzed above, we measured the long-term operational stability of a light-converting device prepared with PNCs dispersed in DCM, being stable under continuous illumination for 480 h at 7.5 V (see Figures 4E and S18), confirming an efficient surface modification by iodide incorporation and defect passivation provided by the Ca mononuclear species. During light converter operation, a blueshift in the PL of the device is visible, indicating the loss of iodide from the active layer, which causes the eventual perovskite deterioration. A comparison of the operational performance of our best light converter with the current state-of-the-art reveals a stability enhancement of approximately ∼ 24-fold relative to prominent perovskite-based devices such as DDAB-CsPbBr_3–*x*_I_*x*_@SiO_2_,^63^ 20-fold higher than CsPbBr_3_ PNCs coated with poly­(maleic anhydride-*alt*-1-octadecene),^64^ and competitive with one of the most stable light-converting device composed of the CsPbBr_3_/mesoporous-SiO_2_ composite, showing a constant PL intensity for 250 h under a lower applied voltage of 3.2 V.^65^ Accordingly, we conclude that the use of DCM as the organic solvent for triggering the halide exchange in the presence of the Ca–I scorpionate complex can be pivotal to process adequate luminescent perovskite inks, even based on another type of halide perovskites, for instance, 3D FAPbX_3_, 2D A_2_SnX_4_, among others, for the fabrication of stable light-emitting devices.

Last, to provide an outlook for future contributions, additional surface engineering strategies could further enhance the stability of the perovskite active layer. In particular, the use of zwitterionic ligands such as sulfobetaines^66^ has shown strong potential for defect passivation due to their ability to simultaneously interact with positively and negatively charged surface sites. Likewise, the deposition of thin inorganic protective layers (e.g., SiO_2_ or Al_2_O_3_)^67–69^ may act as effective barriers against environmental degradation without affecting the PL properties of the perovskite system. In addition, the use of mixed chlorinated organic solvents can contribute to improving film quality and reducing defect density. Together, these approaches represent promising routes to obtain less defective perovskite layers with enhanced protection, ultimately extending the operational stability of the light converters.

## 4 Conclusions

In this work, we have studied the effect of organic solvents on the halide-exchange reaction between green-emitting CsPbBr_3_ and a prominent capping ligand based on a Ca–I scorpionate coordination complex, thereby favoring or inhibiting the formation of multicolor CsPbBr_3–*x*_I_*x*_ MHPs with tunable photophysical properties and surface chemistry. In the absence of CaI_SC_, the polarity of the organic solvent promotes the partial removal of native surface ligands, mediating the particle aggregation and the oxygen incorporation as pivotal ways for stabilizing the structural integrity and improving the PL features of PNCs. After the CaI_SC_ ligand is added, halide substitution is facilitated in the presence of chlorinated solvents, high-polar CHL and DCM being the organic environments where a high density of available iodide species migrates to the perovskite surface, causing the compensation/replacement of halide positions. This is not the case of low-polar DCE, where a low iodide fraction is found, delaying the I-to-Br exchange. Then, in the presence of ACN, halide substitution is restrained, possibly as a consequence of PNCs and CaI_SC_ solvation, restraining iodide diffusion to reach the perovskite lattice. Interestingly, CaI_SC_ monomerization is favored in contact with CsPbBr_3_ PNCs, breaking important Ca–O coordination bonds such as Ca–O-THF interactions and Ca−μ–O–Ca type bridges, modifying the Ca dinuclear ligand system into Ca mononuclear units with less steric hindrance to bind the material surface. Attending to the latter, it was possible to prepare suitable luminescent CaI_SC_-capped CsPbBr_3–*x*_I_*x*_ colloidal inks for the fabrication of stable down-light converters, showing that the PNC active material prepared by using DCM has the longest operational stability up to 480 h. Here, a balance of Ca^2+^, I^–^ ,and O^2–^ fractions during ligand passivation, together with the nature of the organic solvent, provide an insight into the processing of multicolor CsPbBr_3–*x*_I_*x*_ PNCs with enhanced stability to be applied in future light-emitting technologies.

## Supplementary Material



## Notes

Scheme of the CaI_SC_ complex, TEM images, histograms, SAED measurements, XRD patterns, UV–vis, steady-state, and time-dependent PL, DFT calculations, XPS and NMR spectra, tables of the optical properties and surface chemical composition of the CsPbBr_3_ perovskite nanocrystals in the presence and absence of the CaI_SC_ complex, dispersed in different organic solvents, and PL emission of a light converter based on CaI_SC_-capped CsPbBr_3–*x*_I_*x*_ ink in DCM (PDF)
