# Supplementary material for: Stabilized Multicolor CsPbBr3–x I x Nanocrystals via Ca–I Scorpionate Capping for Down-Light Converters
Source: ACS Appl Opt Mater. 2026 Apr 14;4(6):1677–88. doi: 10.1021/acsaom.6c00102 (PMC13317591; doi:10.1021/acsaom.6c00102)
Supplement: Supplementary file 1 [file ot6c00102_si_001.pdf]

Supporting Information
Stabilized Multicolor $CsPbBr_{3-x}I_x$ Nanocrystals *via* Ca-I Scorpionate Capping for Down-Light Converters

Bárbara Nicoleth Vallejos,[1] Ignacio Utreras-Asenjo,[1] Enrique Francés-Poveda,[2] Felipe de la Cruz-Martínez,[2] Juwon Jang,[3] Harumi Correa-Leiva,[1] Víctor Mayorga,[1] Oscar A. Douglas-Gallardo,[1] Francisca Werlinger,[4] Javier Martínez,[5] Camilo Segura,[6] Jhonatan Rodriguez-Pereira,[7,8] Beatriz Julián-López,[9] Seog Joon Yoon,[3] Iván Mora-Seró,[9] Carina Pareja-Rivera,[9,] Agustín Lara-Sánchez[2,]* and Andrés F. Gualdrón-Reyes[1,10]**

[1]Facultad de Ciencias, Instituto de Ciencias Químicas, Universidad Austral de Chile, Isla Teja, Valdivia, 5090000, Chile.
[2]Departamento de Química Inorgánica Orgánica y Bioquímica-Centro de Innovación en Química Avanzada (ORFEO−CINQA), Facultad de Ciencias y Tecnologías Químicas and Instituto Regional de Investigación Científica Aplicada-IRICA, Universidad de Castilla-La Mancha, Ciudad Real, 13071, Spain.
[3]Department of Chemistry, College of Natural Science, Yeungnam University, Gyeongsan, 38541, Republic of Korea.
[4]Departamento de Química Orgánica, Facultad de Química, Universidad de Concepción, Edmundo Larenas 129, Concepción, 4070371, Chile.
[5]Departamento de Química, Facultad de Ciencias, Universidad del Bio-Bio, Concepción, 405138, Chile.
[6]Departamento de Química, Facultad de Ciencias, Universidad de Chile, Las Palmeras 3425, Ñuñoa, Región Metropolitana, 7800003, Chile.
[7]Center of Materials and Nanotechnologies, Faculty of Chemical Technology, University of Pardubice, Nam. Cs. Legii 565, Pardubice, 53002, Czech Republic.
[8]Central European Institute of Technology, Brno University of Technology, Purkyňova 123, Brno, 61200, Czech Republic.
[9]Institute of Advanced Materials (INAM), Universitat Jaume I (UJI), Avenida de Vicent Sos Baynat, s/n, Castellón de la Plana, Castellón, 12071 Spain.
[10]Instituto de Ciencia de los Materiales, Universidad de Valencia, C. José Beltrán 2, Paterna, 46980, Spain.

Corresponding authors: cpareja@uji.es, Agustin.Lara@uclm.es, andres.gualdron@uach.cl

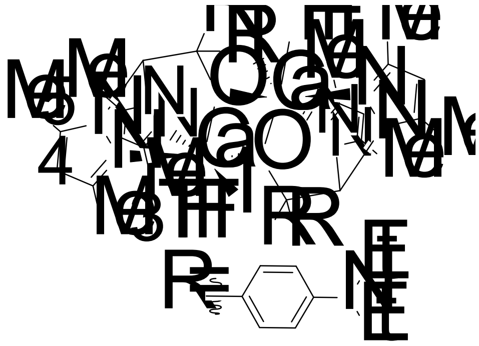

Scheme S1. Structure of the $[CaI(\kappa^3\text{-bpzbdeape})(\mu\text{-O})(thf)]_2$ complex.

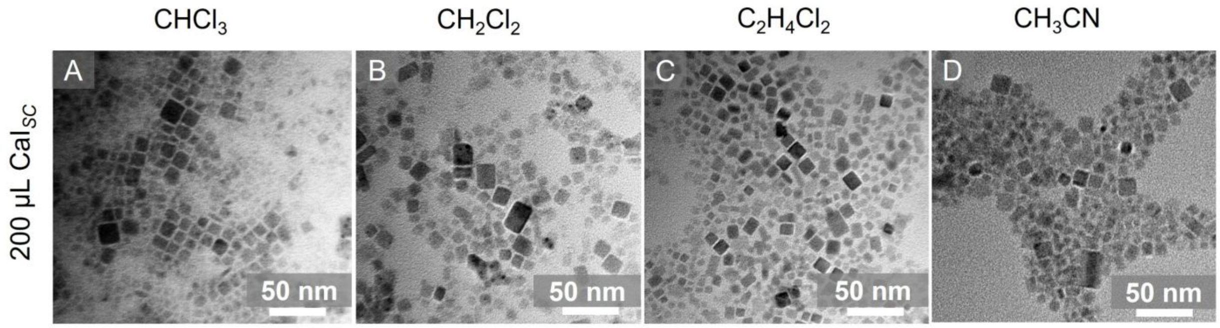

Figure S1. TEM images of $CsPbBr_3$ PNCs after adding 200 µL of CaI_{SC} solution in (A) CHL, (B) DCM, (C) DCE, and (D) ACN.

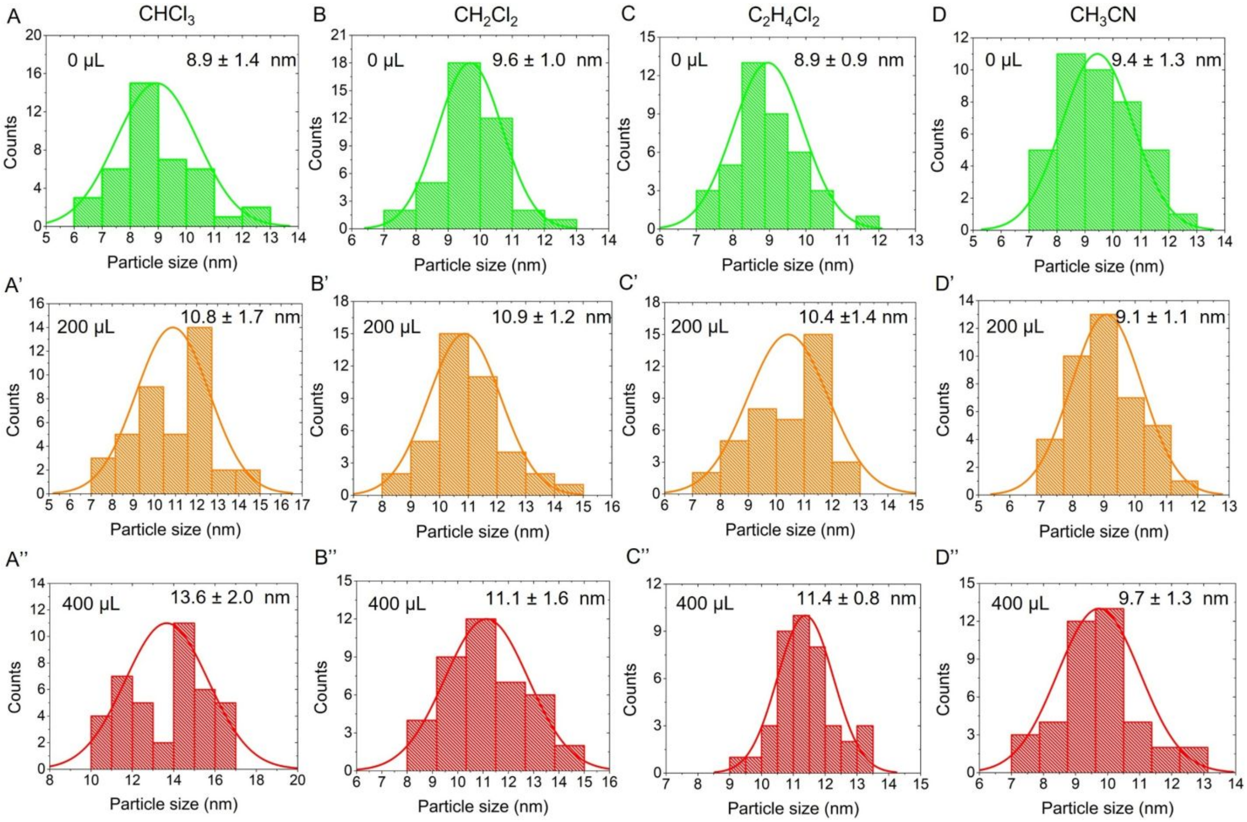

Figure S2. Histograms with particle size distributions obtained from TEM images for $CsPbBr_3$ PNCs after adding different volumes of CaI_{SC}: (A, B, C, D) 0 µL, (A', B', C', D') 200 µL, and (A'', B'', C'', D'') 400 µL, in (A–A'') CHL, (B–B'') DCM, (C–C'') DCE and (D–D'') ACN.

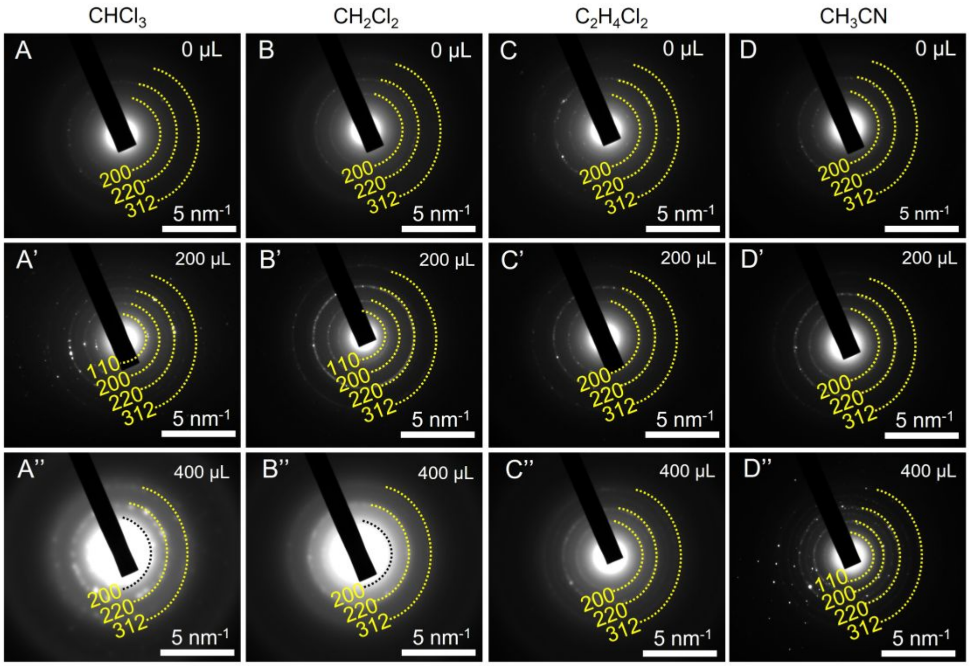

Figure S3. Comparative SAED patterns of $CsPbBr_3$ PNCs after adding different volumes of CaI_{SC}: (A, B, C, D) 0 μL, (A', B', C', D') 200 μL, and (A'', B'', C'', D'') 400 μL, in (A–A'') CHL, (B–B'') DCM, (C–C'') DCE and (D–D'') ACN.

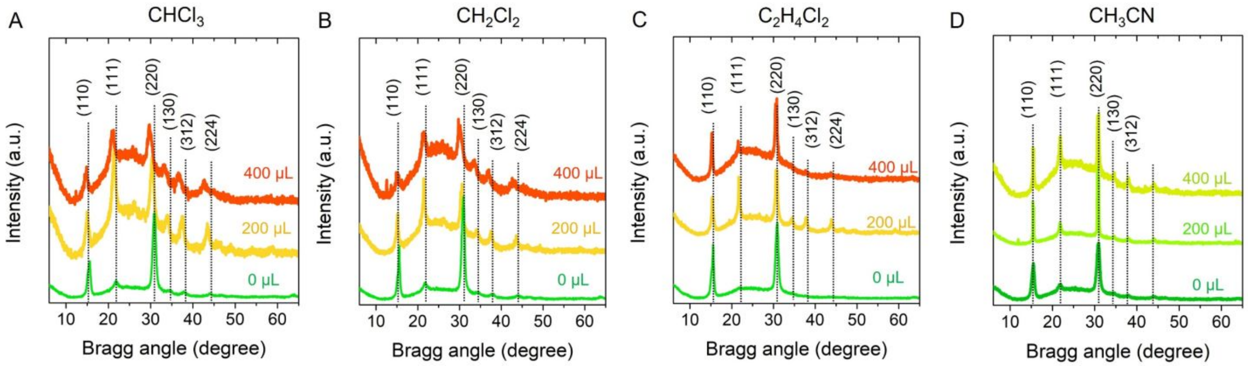

Figure S4. XRD patterns of $CsPbBr_3$ PNCs before and after the ligand-mediated halide exchange reaction using varying volumes of the CaI_{SC} complex in (A) CHL, (B) DCM, (C) DCE, and (D) ACN.

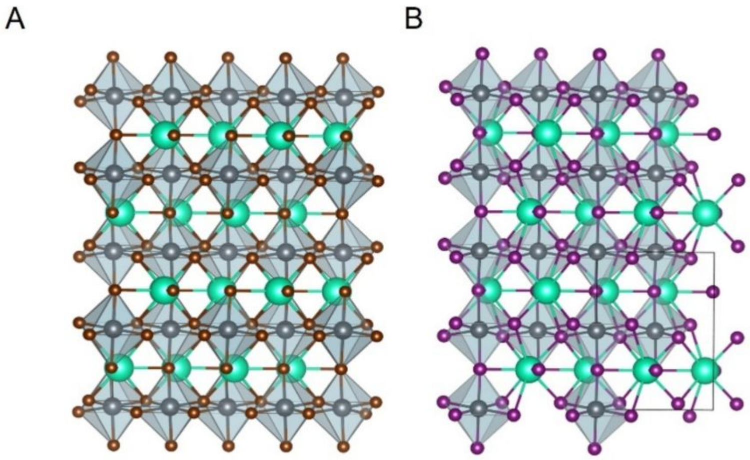

Figure S5. Optimized bulk structures for (A) $CsPbBr_3$ and (B) $CsPbI_3$ perovskite at PBE level. The computed Pb-X bond length was ~3.0 and ~3.2 Å, respectively.

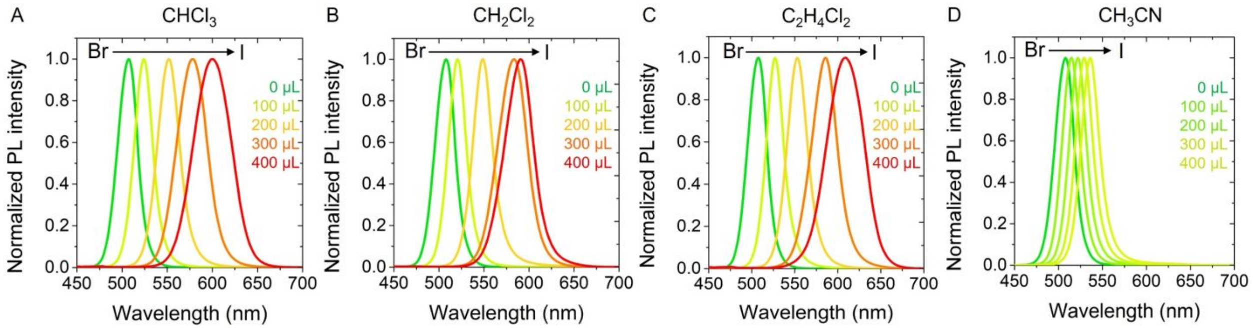

Figure S6. PL spectra of $CsPbBr_3$ PNCs before and after promoting halide exchange with different volumes of CaI_{SC} ligand dissolved in (A) CHL, (B) DCM, (C) DCE, and (D) ACN.

Table S1. Optical properties of the $CsPbBr_3$ PNCs before and after adding different volumes of CaI_{SC} by varying the nature of the organic solvent.

$CsPbBr_3$ + X µL CaI_{SC} in CHL	Absorption edge (nm)	PL peak position (nm)	$CsPbBr_3$ + X µL CaI_{SC} in DCM	Absorption edge (nm)	PL peak position (nm)
0 µL	490	506	0 µL	491	508
100 µL	509	524	100 µL	506	521
200 µL	536	552	200 µL	534	549
300 µL	557	577	300 µL	564	584
400 µL	576	600	400 µL	569	591
**$CsPbBr_3$ + X µL CaI_{SC} in DCE**	**Absorption edge (nm)**	**PL peak position (nm)**	**$CsPbBr_3$ + X µL CaI_{SC} in ACN**	**Absorption edge (nm)**	**PL peak position (nm)**
0 µL	492	507	0 µL	491	507
100 µL	513	528	100 µL	500	514
200 µL	535	552	200 µL	510	521
300 µL	557	585	300 µL	516	528
400 µL	589	609	400 µL	526	535

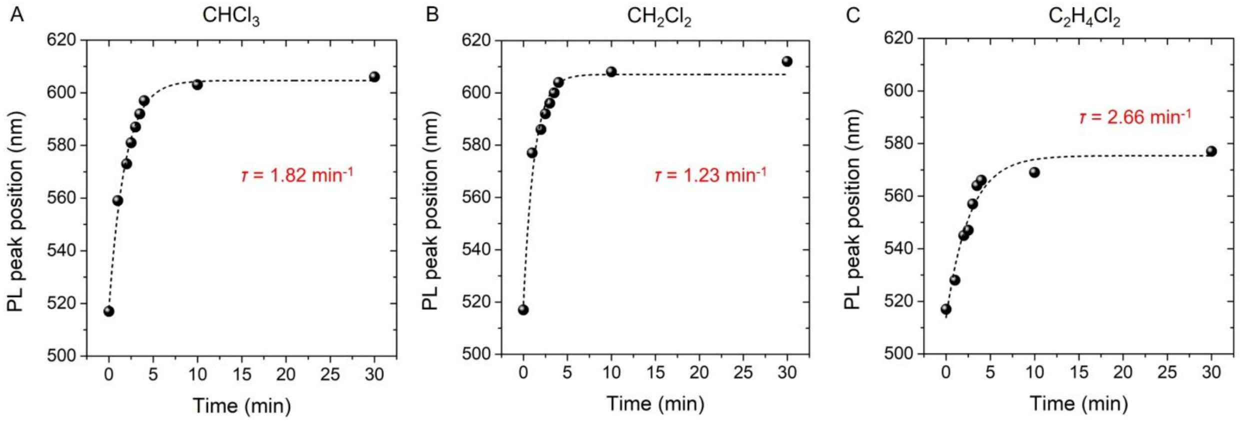

Figure S7. Time-dependence of the maximum PL peak position of CaI_{SC}-capped $CsPbBr_{3-x}I_x$ PNCs in presence of (A) CHL, (B) DCM and (C) DCE. The dashed lines fit well with an empirical pseudo-first-order kinetic model: $y = y_0 + A \cdot exp(-x/\tau)$, where y is the PL peak position at time x, y_0 is the initial PL peak position, A is the amplitude, x is the time and τ is the characteristic time constant for the growth kinetics, respectively.

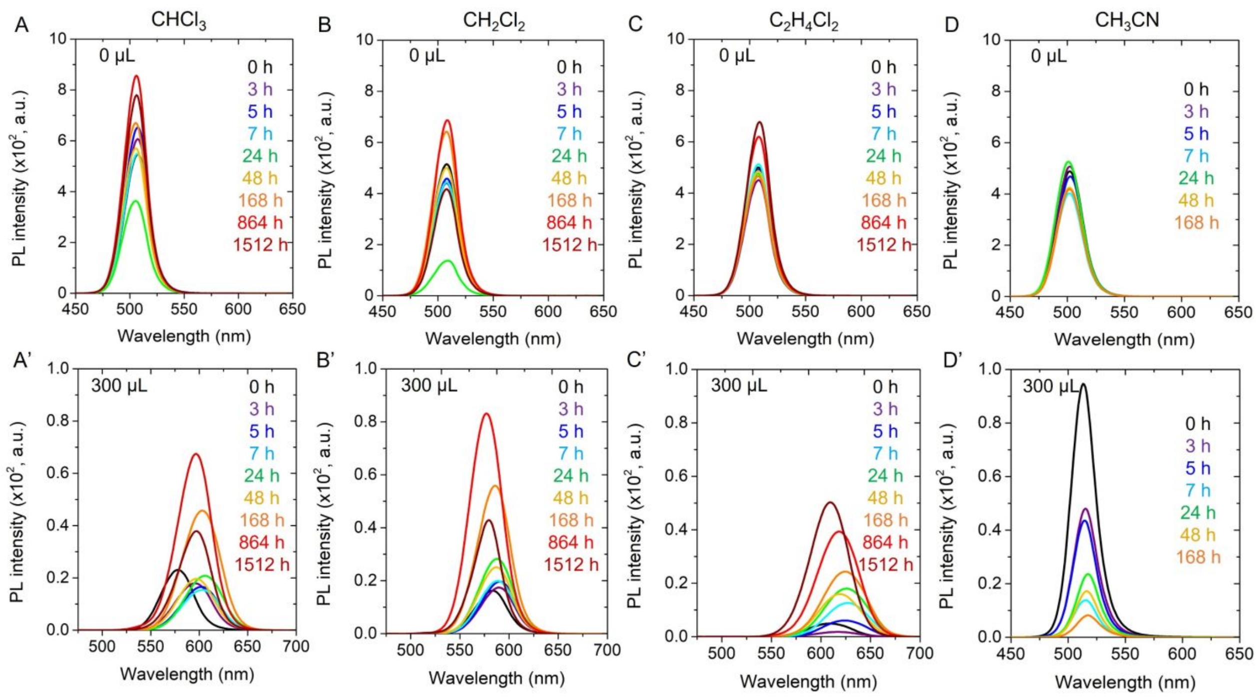

Figure S8. PL stability in function of time of $CsPbBr_3$ PNCs after adding different volumes of CaI_{SC}: (A, B, C, D) 0 µL and (A', B', C', D') 300 µL, in (A, A') CHL, (B, B') DCM, (C, C') DCE and (D, D') ACN.

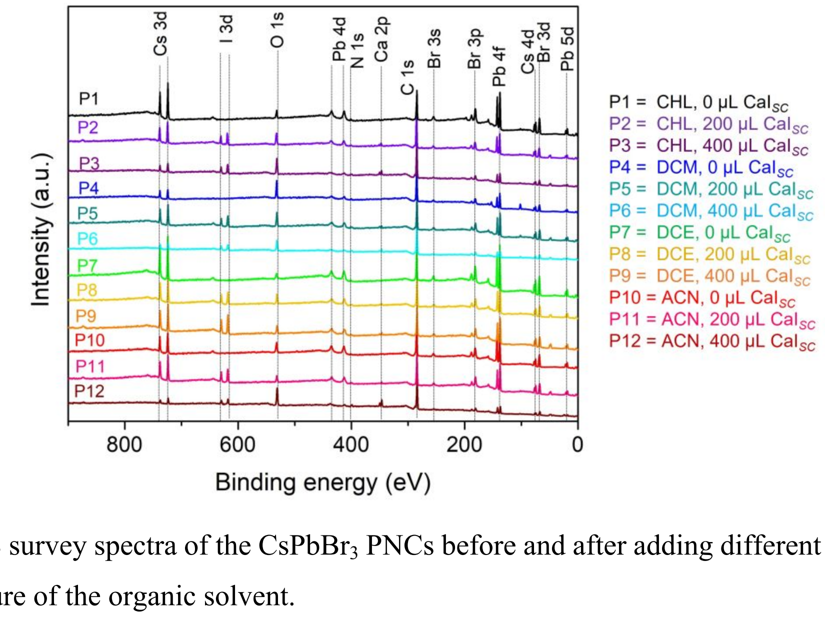

Figure S9. XPS survey spectra of the $CsPbBr_3$ PNCs before and after adding different volumes of CaI_{SC} by varying the nature of the organic solvent.

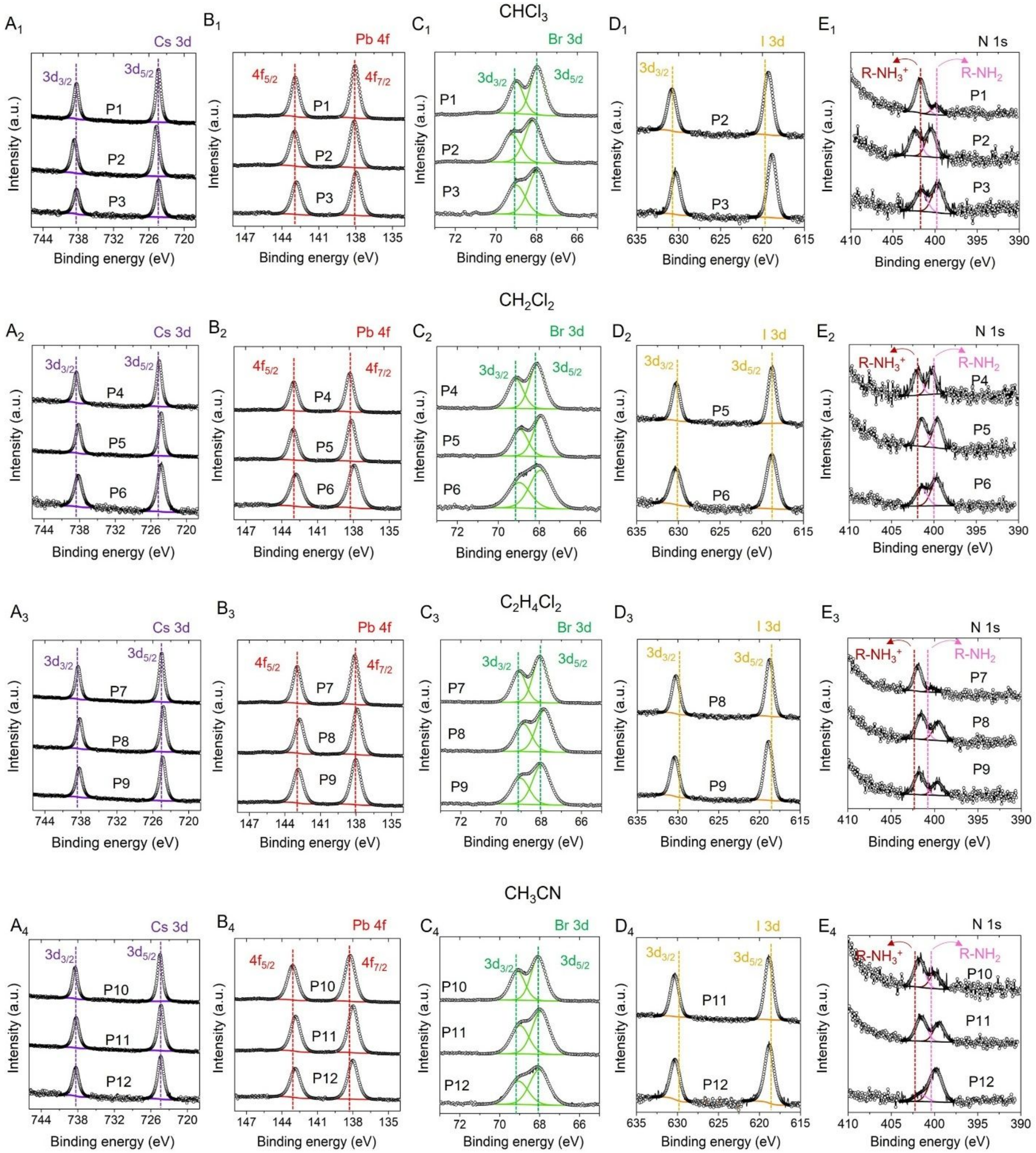

Figure S10. HR-XPS (A_1–A_4) Cs 3d, (B_1–B_4) Pb 4f, (C_1–C_4) Br 3d, (D_1–D_4) I 3d and (E_1–E_4) N 1s spectra of $CsPbBr_3$ PNCs after adding different volumes of CaI_{SC}: (P1, P4, P7, P10) 0 μL, (P2, P5, P8, P11) 200 μL, and (P3, P6, P9, P12) 400 μL in (P1–P3) CHL, (P4–P6) DCM, (P7–P9) DCE and (P10–P12) ACN.

Table S2. Chemical atomic composition of as-prepared $CsPbBr_3$ PNCs before and after adding different volumes of CaI_{SC} by varying the nature of organic solvent, obtained by XPS.

$CsPbBr_3$ – Solvent + X µL CaI_{SC}	Atomic concentration (%)													
	C-(C,H)	C-N	C-O	C=O	(C=O)-OH	C-O	COOH	Pb^{2+}	Br^-	Cs^+	Ca^{2+}	C-NH_2	C-NH_3^+	I^-
P1 – CHL + 0 µL	52.68	6.04	4.43	2.10	1.99	5.97	1.50	4.92	12.42	4.69	-	0.67	2.59	-
P2 – CHL + 200 µL	58.46	7.73	1.87	0.84	2.70	10.19	1.55	2.40	6.37	2.58	1.56	1.23	1.13	1.41
P3 – CHL + 400 µL	63.95	5.14	1.34	0.52	3.40	13.93	0.58	1.09	3.28	1.06	2.63	1.21	0.88	1.00
P4 – DCM+ 0 µL	58.30	12.87	0.89	1.78	1.20	14.14	0.64	1.94	5.18	1.47	-	0.78	0.80	-
P5 – DCM + 200 µL	59.23	6.14	2.05	1.21	2.19	12.63	-	2.54	6.65	2.49	1.26	1.17	1.15	1.29
P6 – DCM + 400 µL	66.59	6.21	1.33	0.95	2.63	12.22	0.26	0.91	2.92	0.91	1.82	1.50	1.01	0.77
P7 – DCE + 0 µL	55.48	5.48	3.01	1.95	0.87	5.46	0.51	5.31	14.49	5.68	-	0.26	1.52	-
P8 – DCE + 200 µL	58.68	6.71	1.81	1.00	1.71	9.92	0.27	3.14	8.12	3.15	1.29	1.12	1.51	1.57
P9 – DCE + 400 µL	58.64	4.94	1.76	1.01	2.38	9.03	0.65	3.35	8.14	3.55	2.01	0.77	1.29	2.49
P10 – ACN + 0 µL	59.10	6.21	3.34	3.15	1.46	9.54	1.10	3.76	7.97	2.44	-	0.68	1.26	-
P11 – ACN + 200 µL	63.64	6.03	1.94	0.64	1.01	6.80	0.15	3.14	7.77	3.36	0.93	1.22	1.59	1.78
P12 – ACN + 400 µL	58.12	7.54	0.95	1.10	3.16	14.67	2.52	0.86	2.72	0.76	4.20	2.18	0.49	0.73

Table S3. Relative concentrations and established atomic ratios for as-prepared $CsPbBr_3$ PNCs before and after adding different volumes of CaI_{SC} by varying the nature of organic solvent, obtained by XPS.

$CsPbBr_3$ – Solvent + X µL CaI_{SC}	Relative concentration					Ratios		
	Cs	Pb	Br	Ca	I	O/(O+Br)	O/(O+Ca)	I/(Br+I)
P1 – CHL + 0 µL	0.95	1.00	2.55	-	-	0.38	-	-
P2 – CHL + 200 µL	1.08	1.00	2.68	0.66	0.60	0.64	0.88	0.18
P3 – CHL + 400 µL	0.97	1.00	3.03	2.44	0.94	0.81	0.85	0.24
P4 – DCM+ 0 µL	0.75	1.00	2.67	-	-	0.74	-	-
P5 – DCM + 200 µL	0.98	1.00	2.62	0.51	0.52	0.66	0.91	0.17
P6 – DCM + 400 µL	1.01	1.00	3.30	1.99	0.88	0.81	0.88	0.21
P7 – DCE + 0 µL	1.11	1.00	2.73	-	-	0.29	-	-
P8 – DCE + 200 µL	0.99	1.00	2.60	0.44	0.51	0.55	0.88	0.16
P9 – DCE + 400 µL	1.05	1.00	2.46	0.60	0.75	0.54	0.83	0.23
P10 – ACN + 0 µL	0.65	1.00	2.12	-	-	0.57	-	-
P11 – ACN + 200 µL	1.07	1.00	2.50	0.30	0.58	0.47	0.88	0.19
P12 – ACN + 400 µL	0.90	1.00	3.27	5.00	0.89	0.86	0.80	0.21

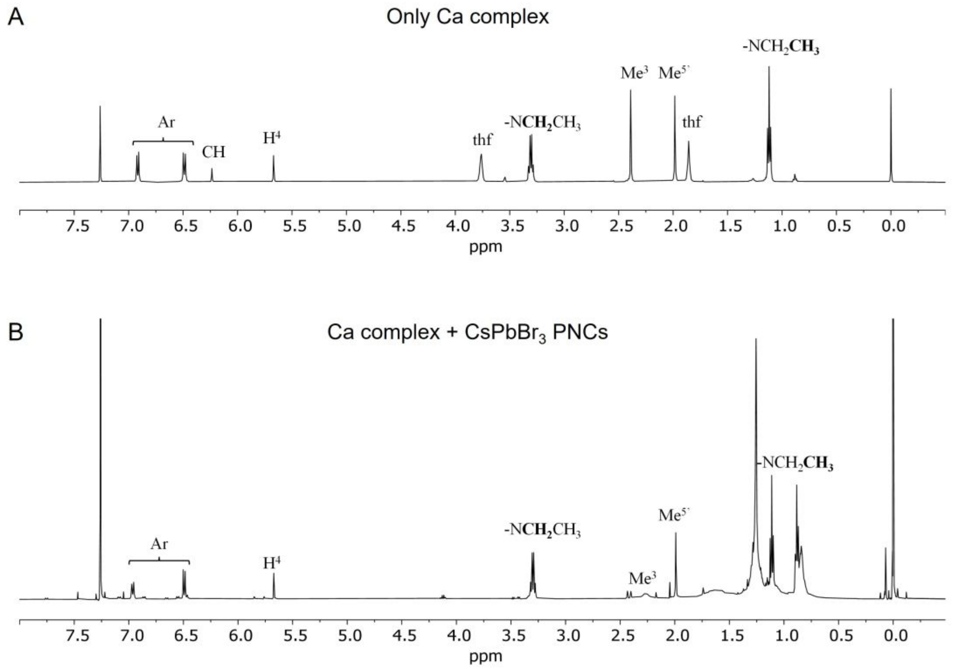

Figure S11. (A) ^{1}H-NMR spectrum of [CaI(κ^3-bpzbdeape)(μ-O)(thf)]$_2$] in $CDCl_3$; (B) ^{1}H-NMR spectrum of CaI$_{SC}$ with $CsPbBr_3$ PNCs in $CDCl_3$.

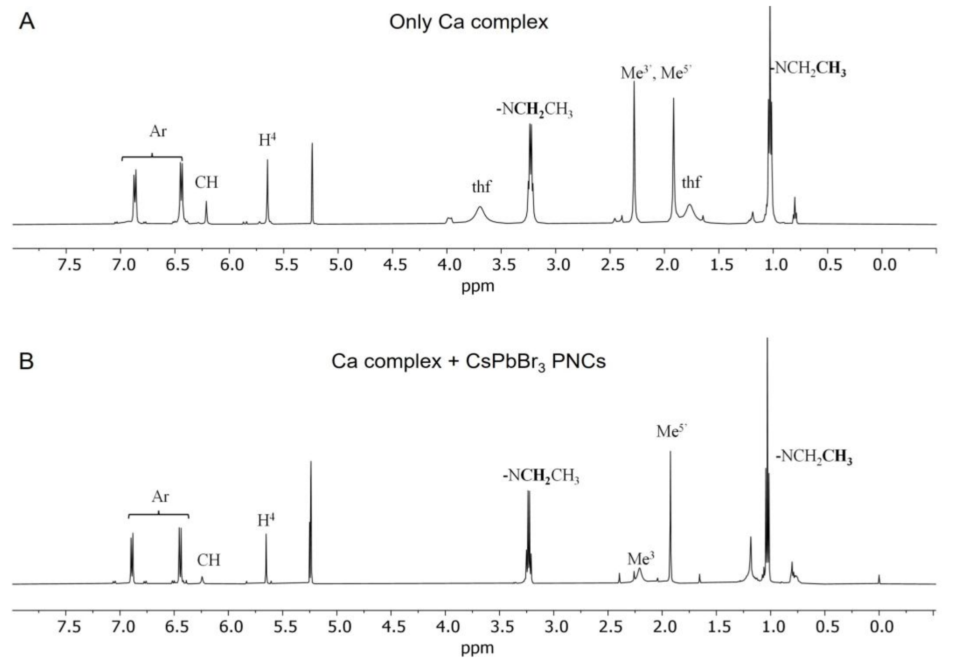

Figure S12. (A) ^{1}H-NMR spectrum of [CaI(κ^3-bpzbdeape)(μ-O)(thf)]$_2$] in $CDCl_3$; (B) ^{1}H-NMR spectrum of CaI$_{SC}$ with $CsPbBr_3$ PNCs in CD_2Cl_2.

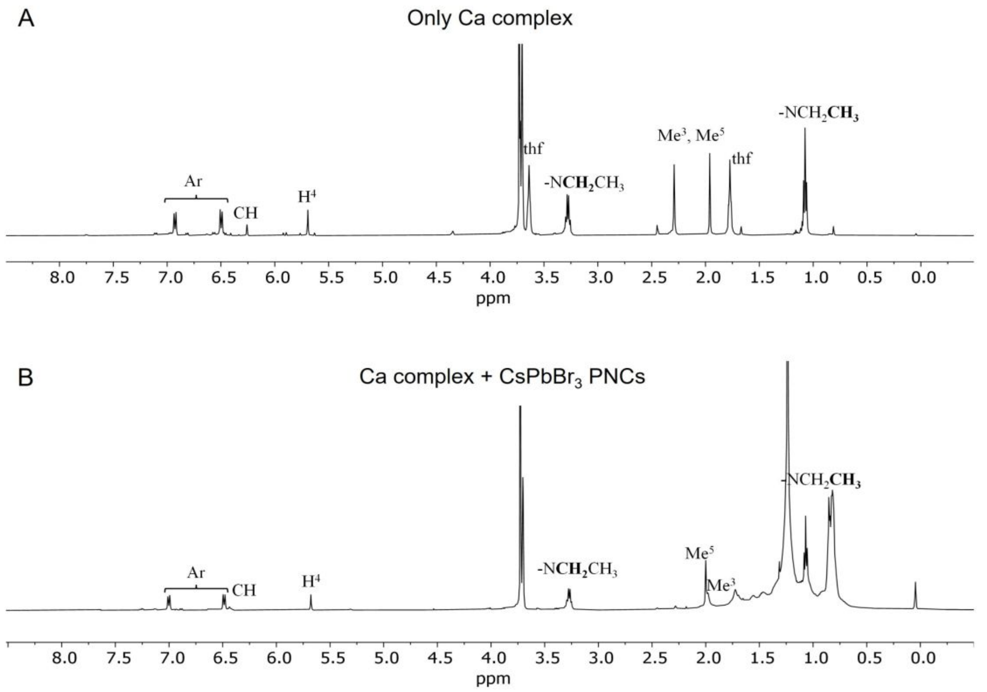

Figure S13. (A) ^{1}H-NMR spectrum of [CaI(κ^3-bpzbdeape)(μ-O)(thf)]$_2$] in $CDCl_3$; (B) ^{1}H-NMR spectrum of CaI_{SC} with $CsPbBr_3$ PNCs in $C_2D_4Cl_2$.

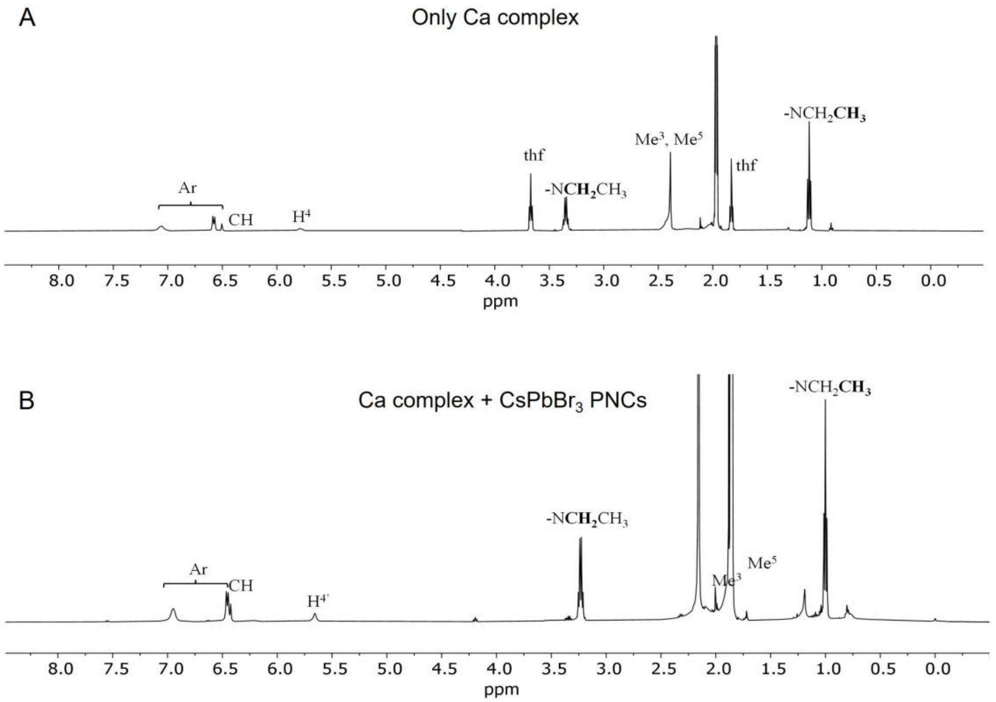

Figure S14. (A) ^{1}H-NMR spectrum of [CaI(κ^3-bpzbdeape)(μ-O)(thf)]$_2$] in $CDCl_3$; (B) ^{1}H-NMR spectrum of CaI_{SC} with $CsPbBr_3$ PNCs in CH_3CN.

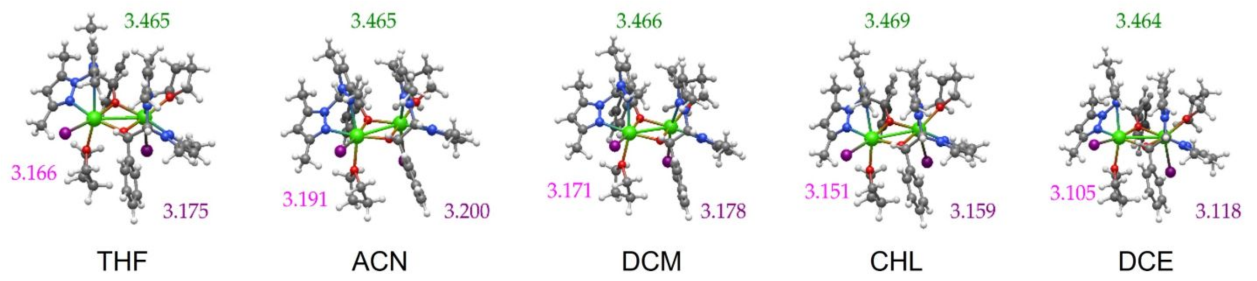

Figure S15. Optimized structures of CaI_{SC} on different organic solvents (THF, ACN, DCM, CHL, DCE) at DFT r^2-SCAN-3c level employing SMD implicit solvent. Selected bond distances are highlighted: Ca-Ca (green), ^{1}Ca-I (violet), and ^{2}Ca-I (pink), respectively. All distances are expressed in Å.

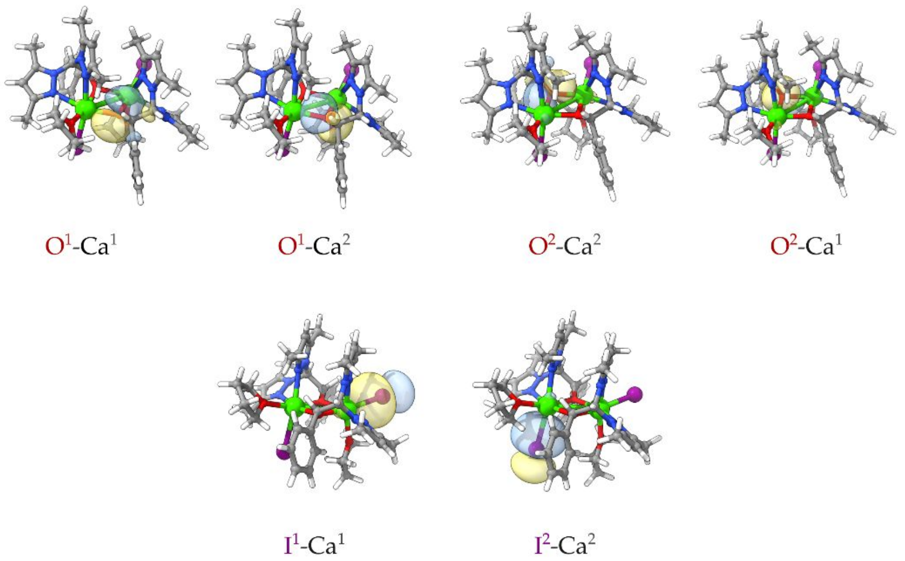

Figure S16. Bond analysis based on Pipek-Mezey (PM) localization method at r^2-SCAN-3c level. The first row shows the Ca-O bonding overlap involving Ca-O-Ca bridge along with Ca-I interaction on the second row with both iodide anions.

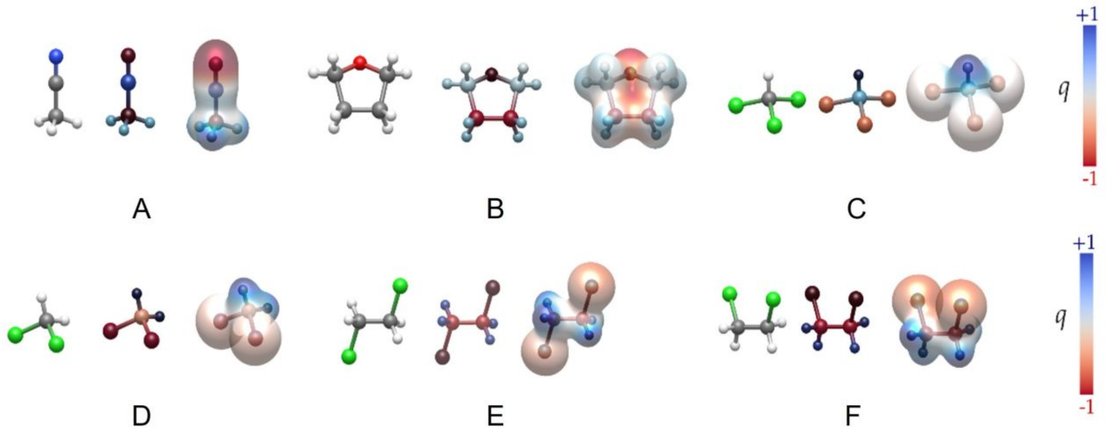

Figure S17. Optimized structure, MBIS partial charge analysis and electrostatic potential map computed at r^2-SCAN-3c level for (a) ACN, (b) THF, (c) CHL, (d) DCM, (e) *E*-DCE, (f) Z-DCE.

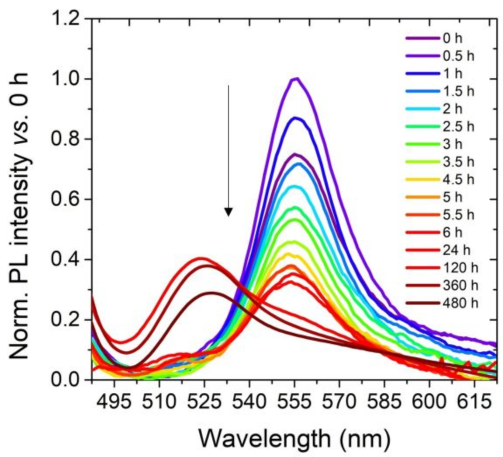

Figure S18. PL spectra of polymeric luminescent down-light converters using as-prepared CaI_{SC}-capped $CsPbBr_{3-x}I_x$ ink in DCM under continuous operation for 480 h.
